# Mesenchymal stem cells shift the pro-inflammatory phenotype of neutrophils to ameliorate acute lung injury

**DOI:** 10.1186/s13287-023-03438-w

**Published:** 2023-08-08

**Authors:** Bing Feng, Xudong Feng, Yingduo Yu, Haoying Xu, Qingqing Ye, Ruitian Hu, Xinru Fang, Feiqiong Gao, Jian Wu, Qiaoling Pan, Jiong Yu, Guanjing Lang, Lanjuan Li, Hongcui Cao

**Affiliations:** 1 State Key Laboratory for the Diagnosis and Treatment of Infectious Diseases, The First Affiliated Hospital, Zhejiang University School of Medicine, 79 Qingchun Rd., Hangzhou, 310003 China; 2 Jinan Microecological Biomedicine Shandong Laboratory, Jinan, 250117 Shandong China; 3 National Clinical Research Center for Infectious Diseases, 79 Qingchun Rd., Hangzhou, 310003 China; 4 Key Laboratory of Diagnosis and Treatment of Aging and Physic-Chemical Injury Diseases of Zhejiang Province, 79 Qingchun Rd, Hangzhou, 310003 China; 5 Department of Chemistry, Duke University, 124 Science Drive, Durham, NC 27708 USA

**Keywords:** Mesenchymal stem cell, Acute lung injury, Neutrophil, Single-cell RNA sequencing, Mouse model

## Abstract

**Background:**

Mesenchymal stem cell (MSC) treatment plays a major role in the management of acute lung injury (ALI), and neutrophils are the initial line of defense against ALI. However, the effect of MSCs on neutrophils in ALI remains mostly unknown.

**Methods:**

We investigated the characteristics of neutrophils in lung tissue of ALI mice induced by lipopolysaccharide after treatment with MSCs using single-cell RNA sequencing. Neutrophils separated from lung tissue in ALI were co-cultured with MSCs, and then samples were collected for reverse transcription-polymerase chain reaction and flow cytometry.

**Results:**

During inflammation, six clusters of neutrophils were identified, annotated as activated, aged, and circulatory neutrophils. Activated neutrophils had higher chemotaxis, reactive oxygen species (ROS) production, and nicotinamide adenine dinucleotide phosphate (NADPH) oxidase scores than aged neutrophils. Circulatory neutrophils occurred mainly in healthy tissue and were characterized by higher expression of *Cxcr2* and *Sell*. Activated neutrophils tended to exhibit higher expression of *Cxcl10* and *Cd47*, and lower expression of *Cd24a*, while aged neutrophils expressed a lower level of *Cd47* and higher level of *Cd24a*. MSC treatment shifted activated neutrophils toward an aged neutrophil phenotype by upregulating the expression of CD24, thereby inhibiting inflammation by reducing chemotaxis, ROS production, and NADPH oxidase.

**Conclusion:**

We identified the immunosuppressive effects of MSCs on the subtype distribution of neutrophils and provided new insight into the therapeutic mechanism of MSC treatment in ALI.

**Supplementary Information:**

The online version contains supplementary material available at 10.1186/s13287-023-03438-w.

## Introduction

Acute lung injury (ALI) is a clinical syndrome characterized by bilateral pulmonary infiltrates, severe hypoxemia, and noncardiogenic pulmonary edema. The most severe form of ALI is acute respiratory distress syndrome (ARDS). More than 10% of ARDS patients worldwide are admitted into intensive care units (ICUs), and severe ARDS has a mortality rate as high as 46% [1]. Despite decades of researches and numerous clinical trials, there are still no effective drugs for treating ARDS. Although several causes of ALI have been identified, including sepsis [2], transfusion [3], and trauma [4], in general ALI is the product of a disturbed inflammatory response that includes the accumulation of activated neutrophils. Many studies have elucidated the critical role of neutrophils in lung tissue during ALI [5–7]. Dysregulated neutrophilic inflammation can destroy lung endothelial and epithelial cells [8]. Activated neutrophils exhibit robust chemotaxis and adhesion, resulting in structural destruction, pulmonary circulation disruption and limitation of gaseous exchange when they rush into the lung tissue [2, 8]. Depletion of neutrophils reduces the severity of lung injury in mice [9]. Summers et al. propose that the therapeutic goal for neutrophil-associated ALI should be to reduce the adverse effects caused by activated neutrophils without damaging host immune defense [10]. Mesenchymal stem cells (MSCs) play the role of an immune regulator in acute liver injury [11], Crohn’s disease [12], and other diseases. MSCs also exert powerful therapeutic effects in ALI through immune regulation [13–16]. Since the COVID-19 pandemic broke out worldwide, numerous clinical trials investigating MSC therapy have been conducted, which have shown promising results for the treatment of ARDS [17–19]. The mechanism underlying the effect of MSCs on macrophages during inflammation has been well studied. MSCs reduce the number of M1 macrophages and dendritic cells (DCs), which inhibits antigen presentation [20]. MSCs produce several anti-inflammatory factors, such as prostaglandin E2 (PGE2) [21], TSG-6 [22], and insulin-like growth factor I (IGF1) [23], to promote the differentiation of alveolar macrophages into an M2 anti-inflammatory phenotype. Studies investigating the effects of MSCs on neutrophils are lacking, but generally it is thought that MSCs can reduce inflammation and enhance the phagocytosis of neutrophils [24–26]. Su et al. report that conditioned medium from MSCs induces lung tissue neutrophil apoptosis in lipopolysaccharide (LPS)-induced ALI [24]. MSCs can enhance neutrophil phagocytosis function and clearance of bacteria [25]. Another study shows that exosomes and conditioned media from MSCs prolong normal neutrophil lifespan and phagocytosis function [26]. LPS-induced ALI has the classic pathological characteristics of ARDS, including infiltration of neutrophils and accumulation of protein-rich fluid in lung tissue [27, 28]. Neutrophils are markers for the therapeutic effects of MSCs in many cases [20, 29, 30]. Here, we perform a high-dimensional analysis of the heterogeneity and phenotypic variety of neutrophils that have infiltrated into lung tissue treated with MSCs using single-cell RNA sequencing (scRNA-Seq). To our knowledge, this is the first report on neutrophil heterogeneity and phenotypic changes in transcriptional and biological functions during LPS-induced ALI after MSC treatment. Characterization of lung neutrophil populations, and their phenotypes and functions, may promote understanding of the mechanisms underlying the immunoregulatory effects of MSCs in ALI, thus providing new insight into MSC therapy for ALI patients.

## Methods and materials

### Animal models of ALI and MSC treatment

All animal experiments were performed with the approval of the Ethics Committee of The First Affiliated Hospital of Zhejiang University (No: 2015-130). 6–8-week-old specific pathogen-free C57BL/6 male mice were utilized as models. All animals were anesthetized with isoflurane (95% oxygen and 5% isoflurane, RWD Inc., Shenzhen, China) inhalation and were euthanized by cervical dislocation when needed. LPS (Sigma-Aldrich, St. Louis, MO, USA) was dissolved in phosphate-buffered saline (PBS), and the solution was administered intratracheally to mice at a concentration of 20 μg LPS per gram of body weight. After 4 h, PBS containing 2% mouse serum, with or without 5 × 10^5^ compact bone-derived MSCs, was given intratracheally to MSC group and LPS group, respectively. There were 5, 10, and 10 mice in PBS group, LPS group, and MSC group, respectively. The characteristics of MSCs are shown in Additional file 1: Fig. S1.

### Detection of cytokine concentrations and neutrophils in bronchoalveolar lavage fluid

Mice among three groups were anesthetized and bronchoalveolar lavage fluids (BALFs) were collected using BD Insyte Intravenous Catheters. 0.6 mL precooled PBS was used for gentle lavage of the lungs twice. The collected BALFs were centrifuged and the supernatants were stored at − 80 °C without thaw-freeze until the experiments. A LEGENDplex mouse cytokine and chemokine panel (BioLegend, San Diego, CA, USA) was used to detect the concentrations of interferon-γ (IFN-γ), interleukin-6 (IL-6) and CXCL1 in BALFs according to the manufacturer instructions. Cells in BALF were stained with Wright-Giemsa reagents (Baso, Zhuhai, China). The number of neutrophils per 200 cells was determined based on morphology.

### Separation of immune cells in lung tissue

Mice were anesthetized and perfused transcardially with PBS until the blood had cleared from the lungs. Lung tissues were homogenized using Mouse Lung Dissociation Kit (Miltenyi Biotec, Bergisch Gladbach, Germany) and GentleMACS™ Dissociator (Miltenyi Biotec). The lung suspensions were then filtered through 70-µm cell strainer (Falcon, New York, MA, USA). After centrifugation, the pellets were resuspended in 5 mL of 40% Percoll (Cytiva, Marlborough, MA, USA) and centrifuged (450 g, 5 min, 4 °C). The pellets were then incubated in red blood cell lysis solution to remove erythrocytes. After washing twice with PBS, the pellets contained isolated lung immune cells.

### Single-cell RNA sequencing

CD45^+^ cells were selected by positive immunoselection using anti-mouse CD45 microbeads (Miltenyi Biotec). Cell viability was detected using trypan blue staining, and samples with viabilities > 95% were used for sequencing. Next, 1 × 10^6^/mL separated lung CD45^+^ immune cells were resuspended in Dulbecco’s PBS (D-PBS) containing 0.04% bovine serum albumin (BSA). Then, the samples were loaded immediately onto a 10× Chromium Controller (10× Genomics, Pleasanton CA, USA). Libraries were constructed using the accompanying kit (10× Genomics). Individual cells were differentiated by barcode, and the expression of transcripts was identified by a unique molecular identifier (UMI). Complementary DNA (cDNA) libraries were sequenced with the Illumina HiSeq 4000 platform (Illumina, San Diego, CA, USA).

### scRNA-seq data processing

Data quality was evaluated using FastQC (Babraham Bioinformatics, Cambridge, UK), and the quality control of data was shown in Additional file 1: Fig. S2. CellRanger (10× Genomics) was adopted to map the sequencing reads based on the mm10 mouse transcriptome (org.Mm.eg.db, Bioconductor; https://bioconductor.org/). Raw data were combined using the R software package (ver. 3.6.1; R Development Core Team, Vienna, Austria), and unless otherwise noted, downstream analyses were performed using the Seurat software package (ver. 3.1.2, Seurat, https://satijalab.org/seurat/). Neutrophil populations were separated from total cells; cells with 200–5000 unique genes and mitochondrial counts < 0.4% were selected for further analysis. The Normalizedata and Scaledata functions were applied using default parameters to remove the differences in sequencing depths across cells. Highly variable genes were evaluated using the FindVariableFeatures function by selecting 2000 genes with the “vst” method. Then, the variable genes were used for principal component analysis (PCA), performed with the RunPCA function. Next, we used the FindNeighbors (dims = 1:15) and FindClusters (resolution = 0.6) functions to cluster the neutrophils. The same principal components (1–15) were selected when using the RunTSNE function for reduction of the dimensions. The top 20 genes for each principal component are shown in Additional file 1: Fig. S3, including many inflammatory genes such as *Ccl3*, *Isg15*, *Cxcl10*, and *Ifitm1*. The FindAllMarker function was used to obtain the marker genes for each cluster. The top 10 marker genes for each cluster are listed in Additional file 1: Table S1. Functional scores were defined by the AddModuleScore function. All of the genes used for scores are listed in Additional file 1: Table S2. A detailed description of other scRNA-Seq data processing is shown in Additional file 1: Supplementary Methods.

### ROS measurement

ROS levels were measured using Reactive Oxygen Species Assay Kit (Beyotime Biotechnology, Shanghai, China). Briefly, separated and cultured lung neutrophils were incubated with 5 μM 2′,7′-Dichlorodihydrofluorescein diacetate (DCFH-DA) for 30 min at 37 °C and then observed using CytoFLEX LX (Beckman Coulter, CA, USA) after washing. The data were analyzed by FlowJo software (Tree Star, OR, USA).

### Western blot analysis

Lung neutrophils separated from LPS group and MSC group on day 3 were solubilized in RIPA lysis buffer (89,900, Thermo Fisher, Rockford, IL, USA) with 1× phosphatase inhibitor (78,420, Thermo Fisher, Rockford, IL, USA) and protease inhibitor (87,786, Thermo Fisher, Rockford, IL, USA). The protein concentrations were detected using BCA kit (Beyotime Biotechnology, Shanghai, China). The western blot was conducted as previously described [31]. NOX2 (SC-130543; Santa Cruz) and GAPDH (D16H11, Cell Signaling Technology) were used for primary antibody, and goat anti-rabbit IgG H&L (HRP) (ab6721; Abcam) and rabbit anti-mouse IgG H&L (HRP) (ab6728; Abcam) were used for secondary antibody. Details are described in the supplementary information (Additional file 1: Supplementary Methods).

### Separation of lung neutrophils and cultured with MSCs

After separating lung immune cells, anti-mouse Ly6G microbeads (Miltenyi Biotec) were used to enrich lung neutrophils. 1 × 10^6^ purified neutrophils were cultured with or without 1 × 10^4^ MSCs in Iscove’s modified Dulbecco’s medium (IMDM) supplemented with 10% fetal bovine serum (FBS), 1% penicillin–streptomycin, and 1 μg/mL LPS for 2 h. For paracrine effects, 1 × 10^6^ neutrophils were cultured in the upper compartment (0.4 μm transwell insert) with or without 1 × 10^5^ MSCs in the lower compartment for 3 h under 1 μg/mL LPS condition. Flow cytometry, ROS measurement, and RT-qPCR were performed after incubation.

### Statistical analysis

Data analyses were performed using GraphPad Prism Software (GraphPad Software Inc., CA, USA), SPSS software package (IBM Corp., Armonk, NY, USA), Seurat R package, and clusterProfiler package. Student’s *t* test was performed to determine significance between two groups. For other comparisons, the statistical methods and corresponding packages were described in the previous section. Data were expressed as the mean ± SEM. *P* < 0.05 was considered to be statistically significant.

### Additional methods

Details on the isolation, purification and differentiation of MSCs, animal models of ALI, lung histology, analysis of differentially expressed genes, scoring of biological processes, analysis of pseudotime trajectory, flow cytometry, immunofluorescence staining, western blot, and RT-qPCR were described in the Additional file 1: Supplementary Methods.

## Results

### Inflammation in ALI was alleviated after MSC treatment

**Fig. 1:**
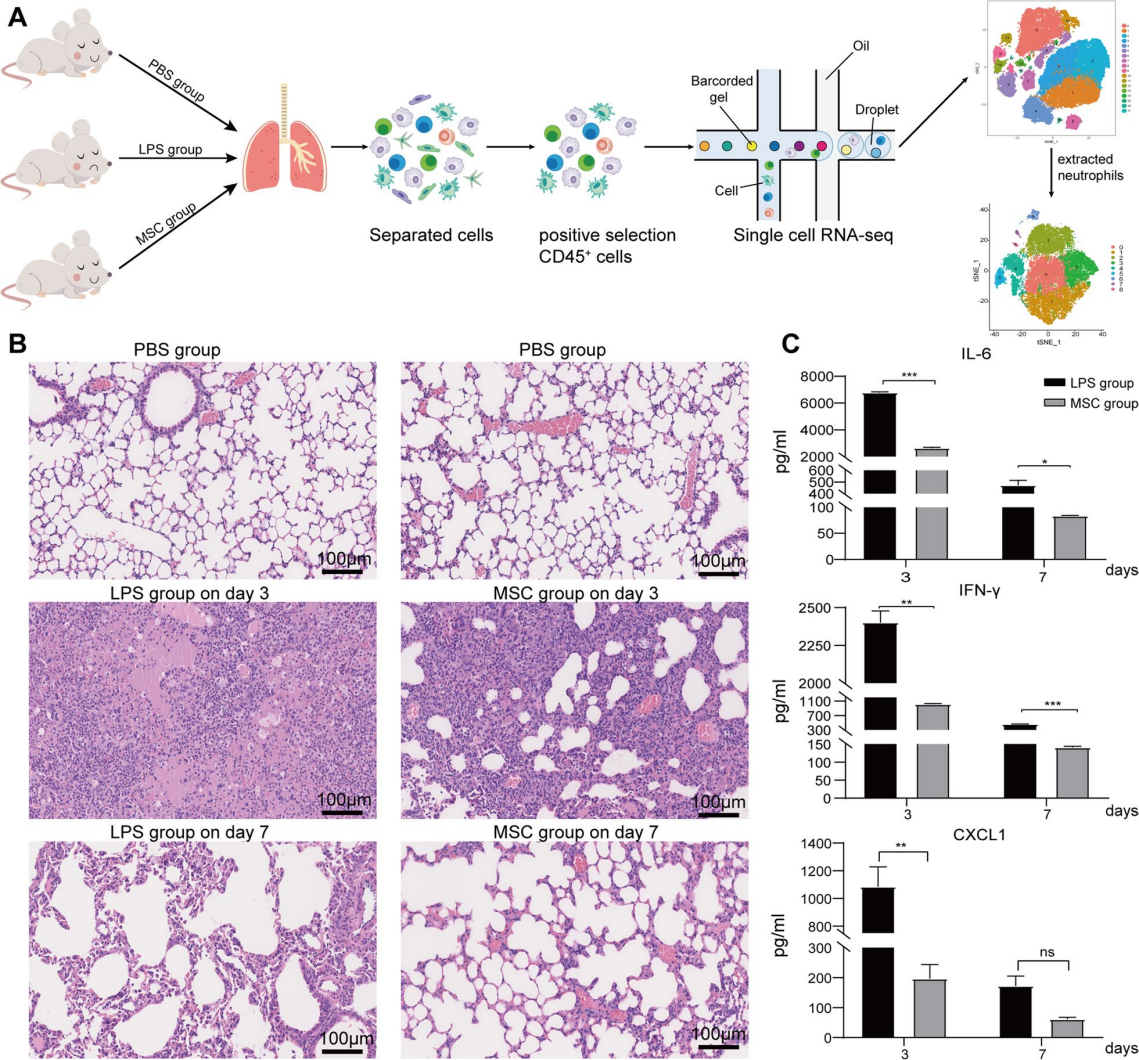
Inflammation was reduced after MSC treatment. **A** Workflow. Lung tissues from mice in each group were collected, and lung immune cells were isolated by *Percoll* density gradient centrifugation. CD45^+^ cells were enriched using CD45 microbeads. scRNA-Seq was used to profile the cells, and after mapping the mouse immune genes, neutrophil subsets were extracted to explore their phenotypes. **B** Representative lung structures from each group. **C** The concentrations of IL-6, IFN-γ, and CXCL1 in BALF over time (*n* = 3). ns, no significance; *, *P* < 0.05; **, *P* < 0.01; ***, *P* < 0.001 unpaired Student’s *t* test

MSCs derived from C57BL/6 mouse compact bone were administrated intratracheally after LPS treatment. The workflow is shown in Fig. 1A. On day 3, hematoxylin and eosin (HE) staining showed that LPS induced cellulose deposition in the alveolar space, infiltration of inflammatory cells, and destruction of normal alveolar structures. On day 7 after LPS treatment, the alveolar structures were almost recovered, the cellulose had been absorbed, and the infiltration of inflammatory cells had been reduced. As expected, recovery was seen as early as day 3 in MSC group. Moreover, the infiltration of inflammatory cells was significantly reduced in MSC group, and the alveolar structures were similar to those of PBS group (Fig. 1B). LPS induced inflammatory conditions in BALF; the level of IL-6 was > 6000 pg/mL, IFN-γ was > 2000 pg/mL, and CXCL1, which could recruit neutrophils, was > 1000 pg/mL. The above cytokines and chemokines levels in BALF were reduced significantly on day 3 in MSC group (Fig. 1C). The concentrations of the acute inflammatory factors IL-6 and IFN-γ were reduced by MSC treatment after 7 days, but there was no significant decrease in the level of CXCL1 (Fig. 1C). Days 3 and 7 after LPS instillation represented the initial inflammatory and recovery phases, respectively.

### Heterogeneity of neutrophils in ALI after MSC treatment

**Fig. 2:**
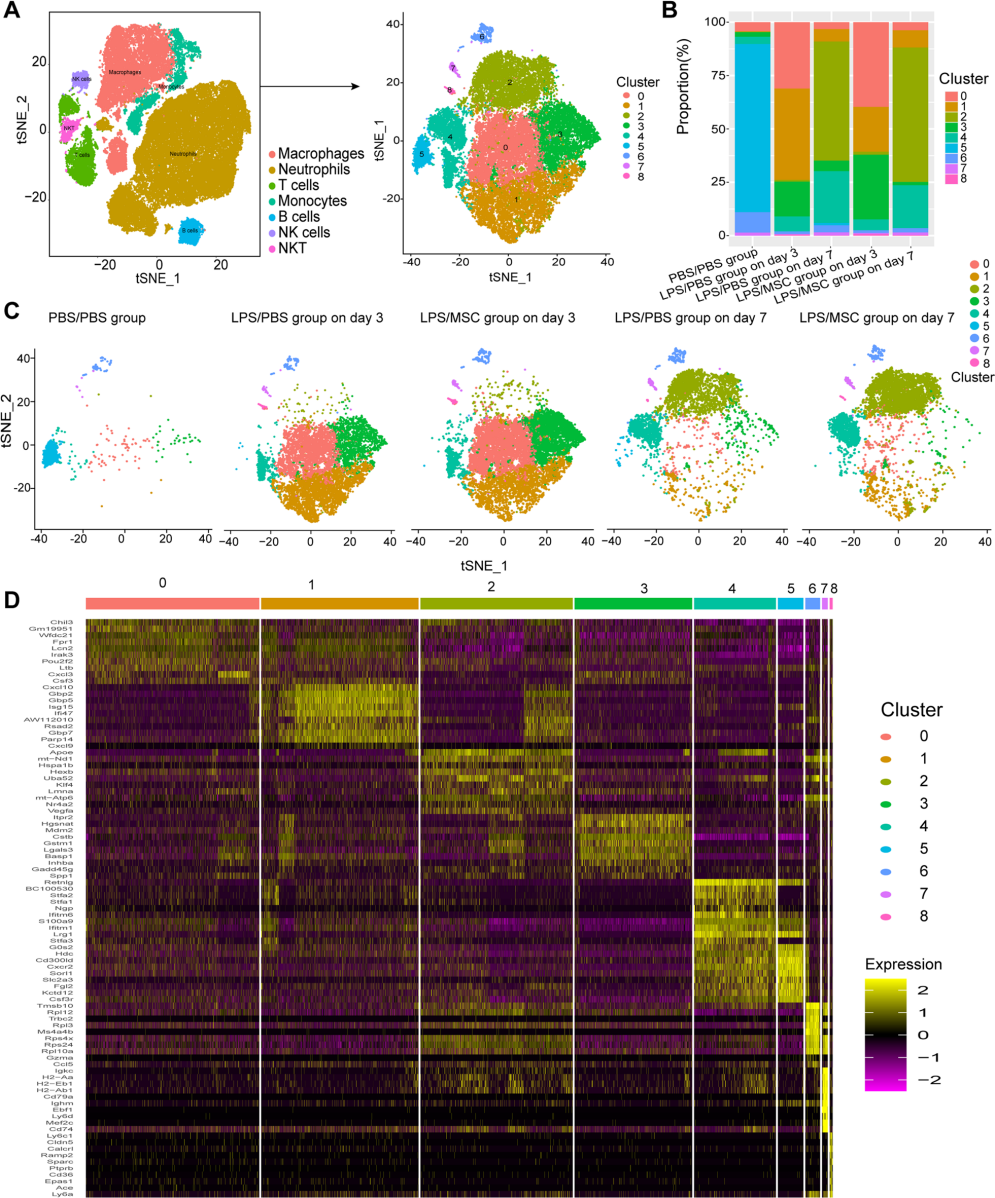
Heterogeneity of neutrophils in ALI after MSC treatment. **A** Neutrophil subsets were extracted from the tSNE map (left). After re-clustering and tSNE dimensionality reduction, we uncovered the heterogeneity among lung neutrophils. The extracted neutrophils were divided into nine different clusters (right). **B** Proportions of the nine neutrophil clusters over time. **C** tSNE map over time. **D** Heatmap of the top 10 marker genes in each cluster

After employing bioinformatics algorithms, unbiased, graph-based clustering identified nine major neutrophil populations (Fig. 2A). The distributions of neutrophils were different among the three groups in the different phases (Fig. 2B, C): in PBS group, the number of neutrophils was initially small, but on day 3 after LPS treatment the number of neutrophils increased dramatically. As shown in Fig. 2B, the proportion of Cluster 1 was reduced, while that of Cluster 3 increased, in MSC group on day 3; however, changes in cell clusters were not obvious between LPS group and MSC group on day 7. Heterogeneity could also be identified based on the marker genes of each cluster (Fig. 2D). The proportions of Clusters 6–8 were relatively small and were characterized by high expression of *Ighm*, *Cd79a,* and *Ms4a4b*, which were likely expressed by B cells. Therefore, these three clusters were excluded from subsequent analyses.

### Features of neutrophils in ALI after MSC treatment

#### Defining neutrophil phenotypes through aging-related genes

**Fig. 3:**
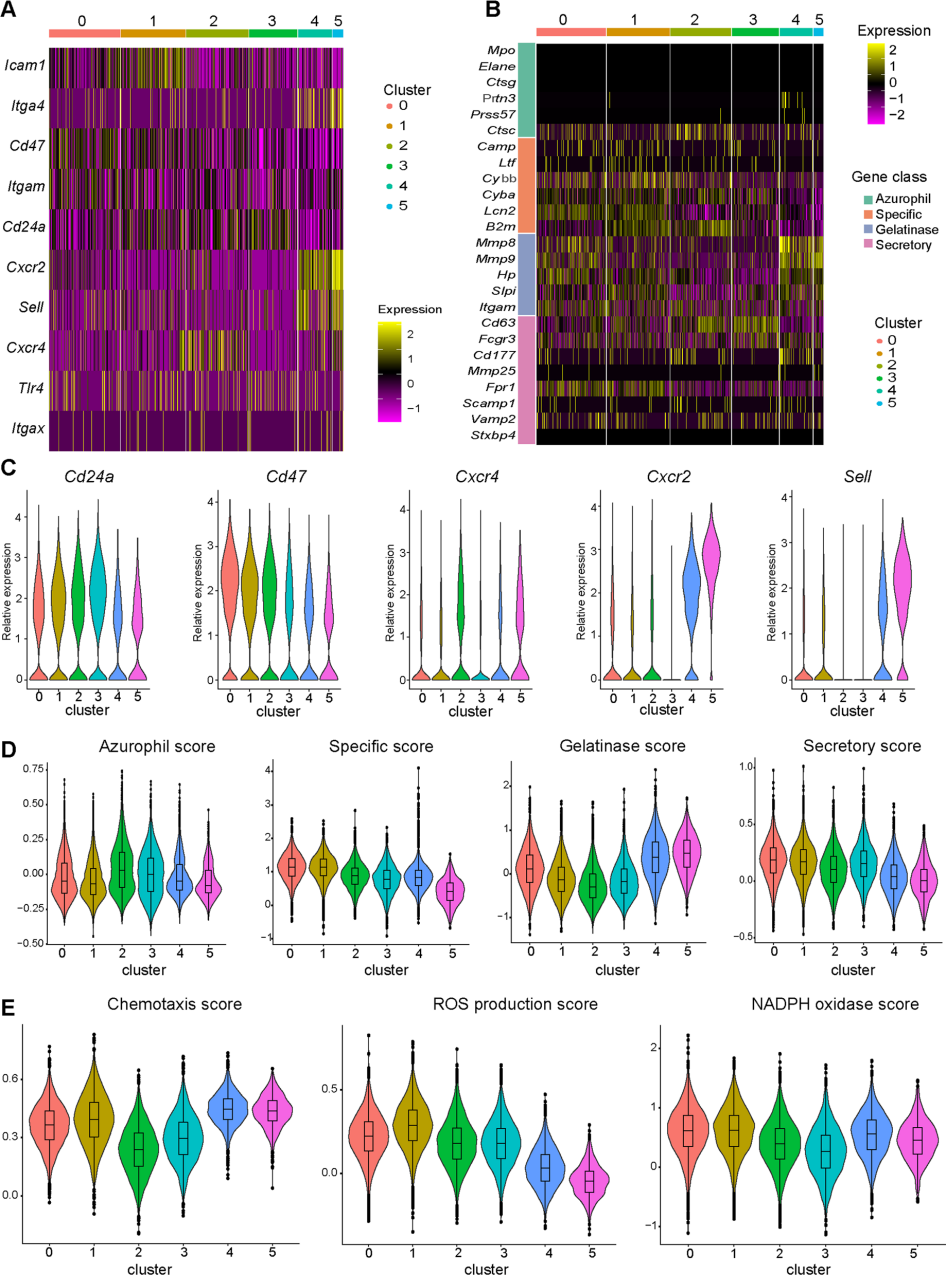
Characterization of neutrophils in ALI after MSC treatment. **A** Heatmap of aging-related genes in neutrophils. **B** Heatmap showing the expression of neutrophil granule-related genes. **C** Violin plots of the relative expression of *Cd24a*, *Cd47*, *Cxcr4*, *Cxcr2,* and *Sell*. **D** Violin plots of azurophil, specific, gelatinase, and secretory scores for the six clusters. **E** Violin plots of chemotaxis, ROS production, and NADPH oxidase scores for the six clusters

Next, the characteristics of these neutrophil populations were studied. Aging-related genes were differentially expressed among the neutrophil populations. *Itga4* (encoding CD49d), *Cxcr2* (encoding CXCR2, a molecule related to neutrophil chemotaxis [32]), and *Sell* (encoding CD62L, a migration molecule) were highly expressed in Clusters 4 and 5 (Fig. 3A). Cluster 5 was the predominant subgroup in the PBS group, and Cluster 4 appeared mainly on day 7 after treatment (Fig. 2B). These data suggested that on day 7 after treatment, there was a trend toward recovery along with the decreased concentrations of IL-6, IFN-γ, and CXCL1. Based on this information, these two clusters were annotated as circulatory neutrophils. In addition, Clusters 0 and 1 increased significantly on day 3 after LPS treatment. Cluster 0 expressed a high level of *Cd47* (encoding a molecule that inhibits the clearance of dead neutrophils) and a low level of *Cd24a* (encoding a molecule that induces apoptosis after being crosslinked) (Fig. 3A). Cluster 1 expressed increased *Cxcl10* (encoding CXCL10) and *Icam1*, so these two clusters were annotated as activated neutrophils. Cluster 2 highly expressed *Cxcr4* (encoding a molecule that regulates neutrophil release from and return to bone marrow). Cluster 3 expressed a high level of *Cd24a* and had lower expression of *Cd47*. Based on Xie and colleagues’ research [33], aged neutrophils exhibit increased expression of CD24 and CXCR4 and reduced expression of CD47, so Clusters 2 and 3 were annotated as aged neutrophils.

#### Expression of granule genes in each cluster

In addition to age-related genes, the expression of granule genes in each cluster was also evaluated. It was not surprising that *Elane, Prtn3,* and *Mpo* expression levels were the same in all clusters (Fig. 3B) as these genes were mainly expressed in neutrophils located in bone marrow [33], whereas the isolated neutrophils were derived from peripheral lung tissue. The activated neutrophils (Clusters 0 and 1) had higher expression of either specific or secretory granule genes. The aged neutrophils (Clusters 2 and 3) displayed lower expression of gelatinase granule genes; Cluster 3 also had lower expression of specific granule genes. The circulatory neutrophils (Clusters 4 and 5) highly expressed gelatinase granule genes such as *Mmp8* and *Mmp9*. The relative expression levels of selected genes in each cluster are shown in Fig. 3C.

The biological functions of these clusters were further analyzed. The aged neutrophils (Clusters 2 and 3) had a higher azurophil score. The activated neutrophils (Clusters 0 and 1) had higher specific and secretory scores, while the circulatory neutrophils (Clusters 4 and 5) had a higher gelatinase score (Fig. 3D). In addition, the aged neutrophils had lower scores for chemotaxis, ROS production, and nicotinamide adenine dinucleotide phosphate (NADPH) oxidase, while the activated neutrophils had higher chemotaxis, ROS production, and NADPH oxidase scores. The circulatory neutrophils had a higher chemotaxis score, probably due to the overexpression of *Cxcr4*, which drived them toward the bone marrow; they also had the lowest ROS production score (Fig. 3E).

#### Pseudotime trajectories of each cluster

**Fig. 4:**
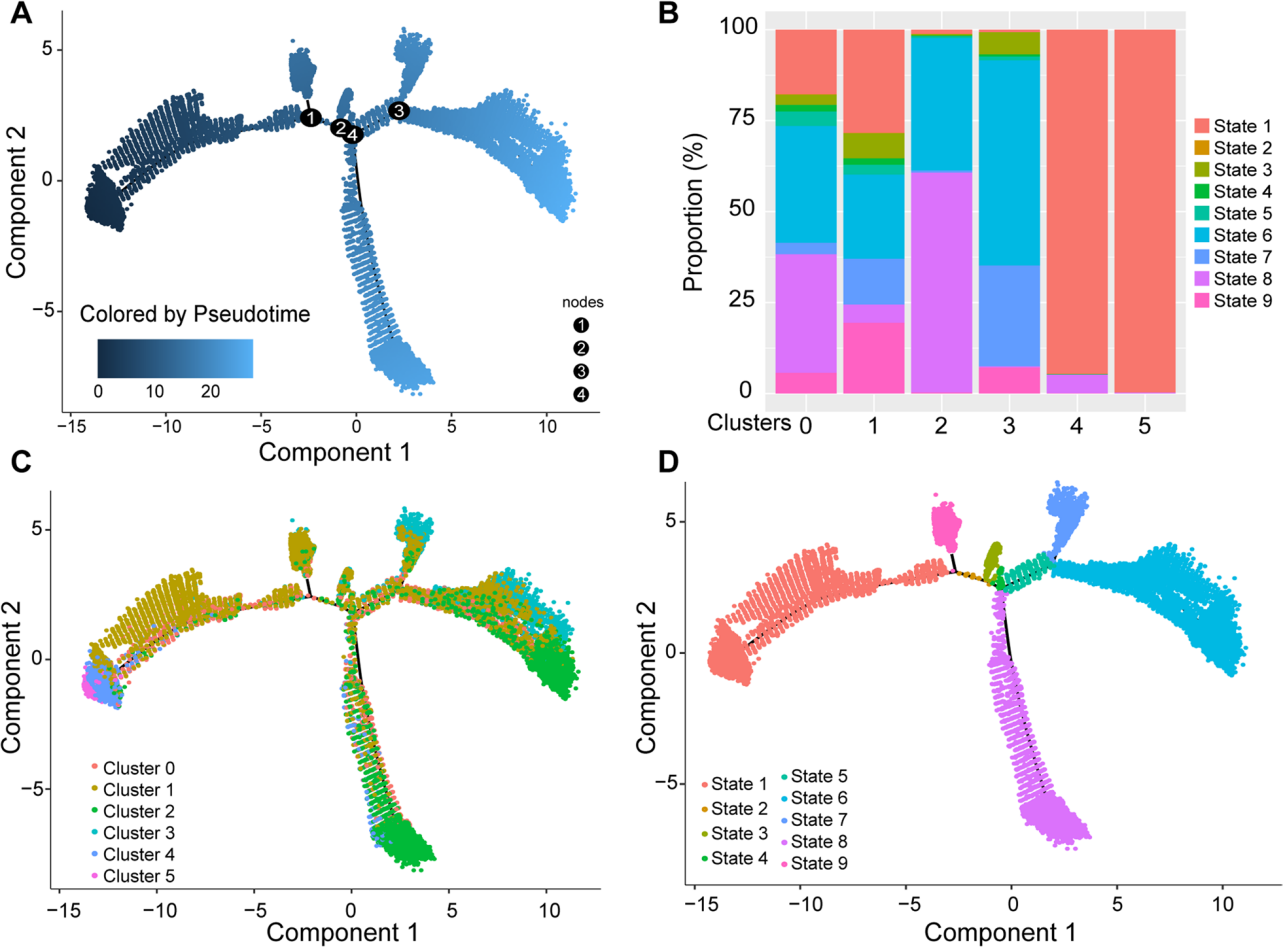
Pseudotime trajectories of lung neutrophils in ALI after MSC treatment. **A** Monocle-guided cell trajectory of neutrophils colored from dark to light blue. The start of pseudotime was indicated by dark blue and the end of pseudotime by light blue. And the nodes represented branches of differentiation trajectories. **B** Analysis of the neutrophil distribution among the nine transcriptional states. Color bar graphs represent the frequency of nine states in each clusters. **C**, **D** Monocle-guided cell trajectory of neutrophils colored by 6 clusters (**C**) and 9 transcriptional states (**D**)

Next, the annotated clusters were characterized using the monocle R package. As expected, the circulatory neutrophils (Clusters 4 and 5) and aged neutrophils (Clusters 2 and 3) were positioned at the opposite ends of the trajectory, that is, circulatory neutrophils were mainly in state 1, while the aged neutrophils were predominantly in states 6–8. The activated neutrophils were unevenly distributed in all states (Fig. 4).

Overall, circulatory neutrophils were found mainly in the PBS group and at the initiation of the pseudotime trajectory. The activated neutrophils showed a more pro-inflammatory phenotype than the aged neutrophils.

### Changes in neutrophil transcription and biological functions on day 3 after MSC treatment

**Fig. 5:**
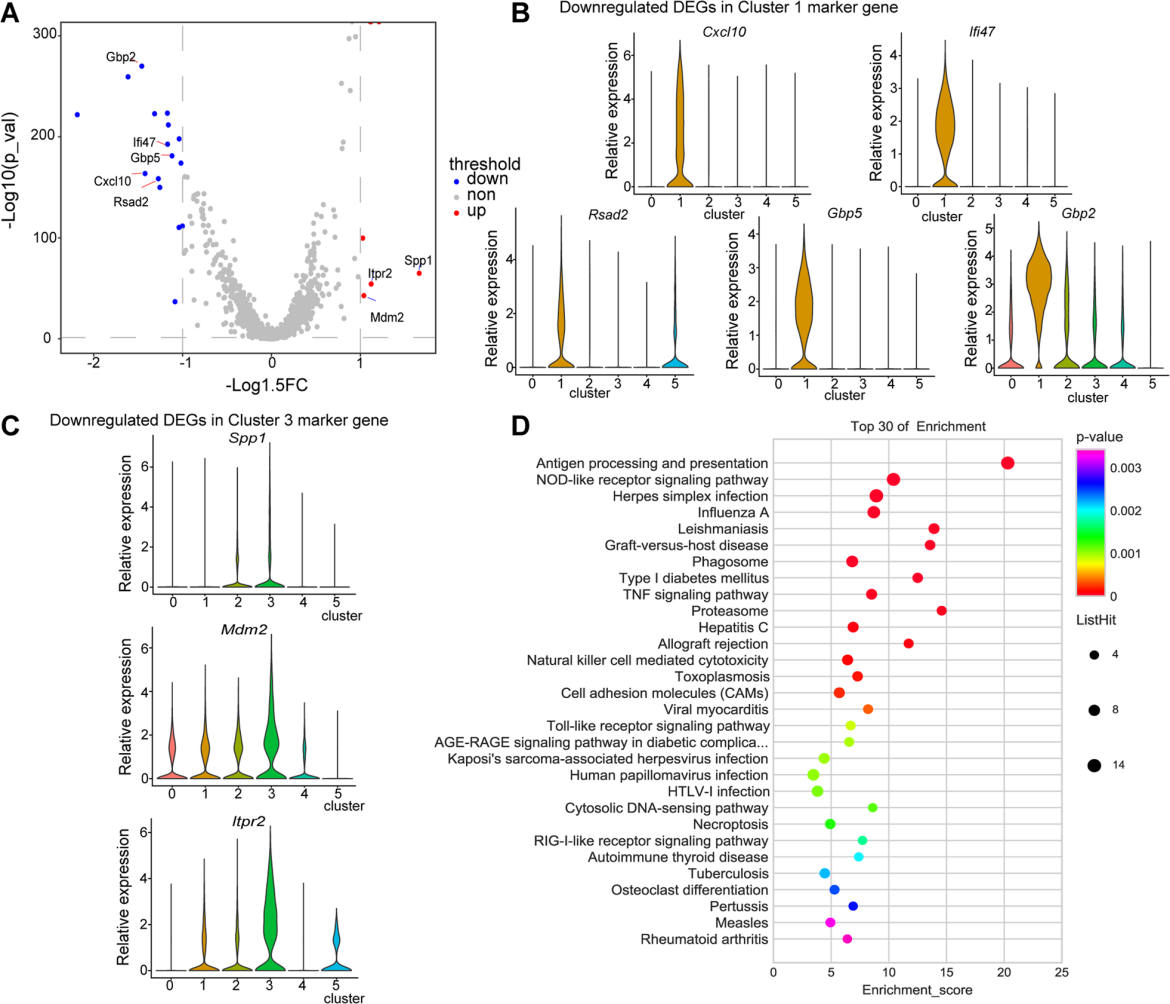
Effects of MSC treatment on lung neutrophil transcriptional changes on day 3. **A** Volcano plot showing the DEGs in LPS versus MSC group. *P* value was calculated using the Wilcoxon test. Genes with *P* value < 0.05 and foldchange > 1.5 were selected. Blue and red dots represented genes downregulated and upregulated by MSCs, respectively. Marker genes for Clusters 1 and 3 were annotated. **B** Violin plots showing the relative expression of downregulated DEGs by MSCs in marker genes of Cluster 1. **C** Violin plots showing the relative expression of upregulated DEGs by MSCs in marker genes of Cluster 3. **D** Bubble chart showing the KEGG enrichment analysis of the top 30 functional DEGs between MSC and LPS groups on day 3 with a foldchange > 1.2 and *P* value < 0.05

After analyzing the differentially expressed genes (DEGs) between MSC and LPS groups on day 3, we discovered that MSC treatment mainly affected Clusters 1 and 3. As shown in Fig. 2B, the size of Cluster 1 was reduced, while that of Cluster 3 was increased on day 3 after MSC treatment. Interestingly, the marker genes for Cluster 1, such as *Cxcl10*, *Ifi47*, *Rsad2*, *Gbp2,* and *Gbp5*, were downregulated by MSCs (Fig. 5A, B), while those for Cluster 3, including *Spp1*, *Mdm2*, and *Itpr2*, were upregulated (Fig. 5A, C). The expression of *Icam1* was also downregulated after MSC treatment*,* which was at a high level in Cluster 1 (Additional file 1: Table S3). The pathways between LPS group and MSC group, based on the downregulated DEGs on day 3, were analyzed by clusterProfiler. The downregulated DEGs were involved in some inflammatory pathways, such as antigen processing and presentation, the nucleotide-binding oligomerization domain (NOD)-like receptor signaling pathway, phagosome and the tumor necrosis factor (TNF) signaling pathway (Fig. 5D).

**Fig. 6:**
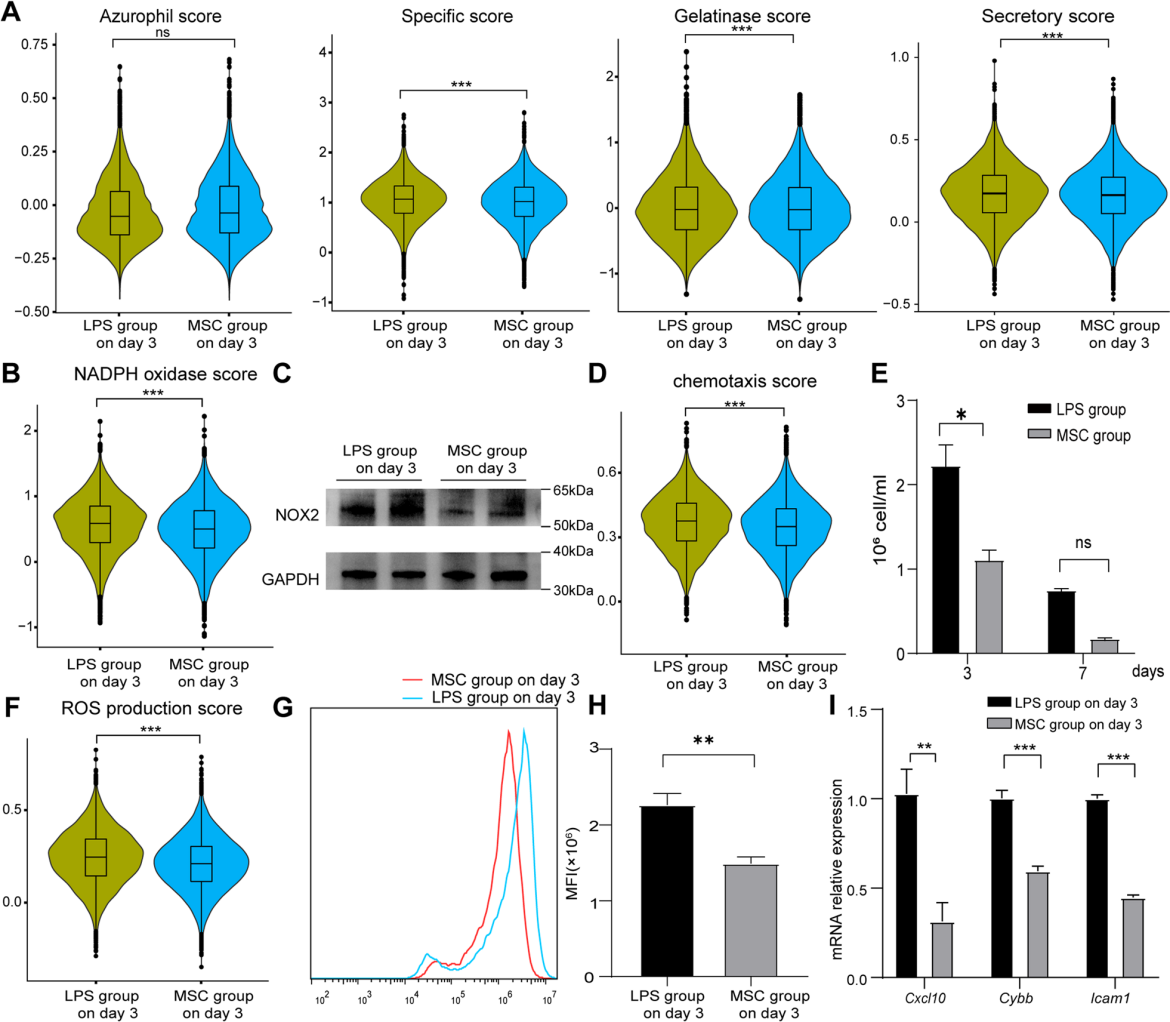
The functional changes of lung neutrophils on day 3 after MSC treatment. Violin plots of azurophil, specific, gelatinase, and secretory scores (**A**); NADPH oxidase score (**B**); chemotaxis scores (**D**); ROS production scores (**F**) on day 3: LPS versus MSC group. **C** NOX2 protein levels in lung neutrophils were detected by WB analysis. Full-length blots were presented in Additional File 1: Figure S7. **E** Absolute numbers of neutrophils (*n* = 3) in lung over time. **G** Relative ROS levels were measured by DCFH-DA probe in separated lung neutrophil. **H** Bar plot showing mean fluorescence intensity (MFI) of DCFH-DA probe (*n* = 4). **I** Bar plots showing the different expression of *Cxcl10*, *Cybb*, and *Icam1* (*n* = 4). ns, no significance; *, *P* < 0.05; **, *P* < 0.01; ***, *P* < 0.001 unpaired Student’s *t* test

Neutrophil functions including the synthesis of granular proteins were inhibited after MSC treatment, apart from azurophil granules (Fig. 6A). NADPH oxidase score was also significantly downregulated by MSCs (Fig. 6B). Western blot analysis showed the level of NOX2 protein, which involved in NADPH oxidase and granule secretion [34], was decreased (Fig. 6C). After analyzing the expression of genes involved chemotaxis, we found that the lung neutrophil chemotaxis score was reduced after MSC treatment (Fig. 6D). At the same time, the number of neutrophils in BALF was reduced significantly after 3 days (Fig. 6E). The ROS production score was downregulated after MSC treatment (Fig. 6F). The lung neutrophil ROS level detected by DCFH-DA probe was decreased significantly (Fig. 6G, H). Biological function related genes, including chemokine gene *Cxcl10*, ROS-related gene *Cybb* (also named NOX2), and cell adhesion-related gene *Icam1,* were validated by RT-qPCR. The results of RT-qPCR showed that the expression of *Cxcl10*, *Cybb,* and *Icam1* was significantly reduced on day 3 after MSC treatment (Fig. 6I).

### Differential characteristics of clusters 1 and 3

Since MSC treatment mainly affected Clusters 1 and 3, we further analyzed the two clusters. An analysis of DEGs between activated neutrophils (Cluster 1) and aged neutrophils (Cluster 3) showed that *Cxcl10* was the most upregulated gene among the activated neutrophils; in addition, other inflammation-related genes such as *Isg15*, *Ifitm1*, *Ifitm3,* and *Ifi47* were also upregulated in Cluster 1. Interestingly, *Cd274* (encoding PD-L1) was upregulated in Cluster 1 (Additional file 1: Fig. S4a, b). The upregulated genes in Cluster 1 were involved in inflammatory pathways, such as the NOD-like receptor, TNF, and Toll-like receptor signaling pathways (Additional file 1: Fig. S4c). The upregulated genes in Cluster 3 were mainly involved in lysosome, spliceosome, and apoptosis pathways (Additional file 1: Fig. S4d). These results demonstrated that Cluster 1 had an activated inflammatory phenotype, while Cluster 3 had an aged phenotype.

### The expression of CD24 on inflammatory neutrophils was upregulated by MSCs

**Fig. 7:**
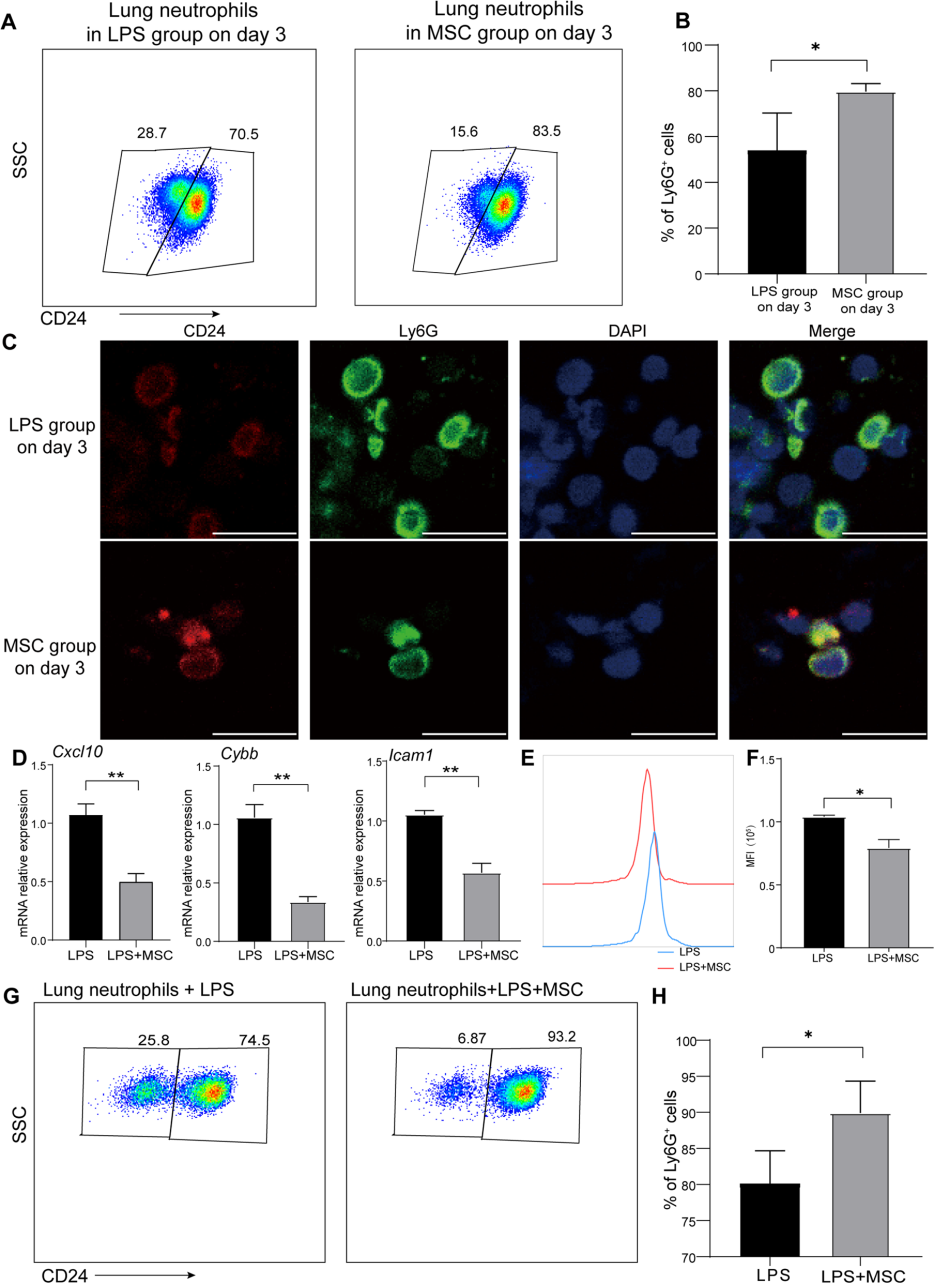
MSC treatment shifted the neutrophil phenotype by upregulating the expression of CD24. **A** Representative flow cytometric dot plots showing the subpopulations of lung neutrophils defined by CD24 expression on day 3 in LPS versus MSC group. **B** Bar plot showing the proportion of CD24^hi^ neutrophils in LPS versus MSC group (*n* = 4). **C** Distribution of lung neutrophils expressed CD24 on day 3 in LPS versus MSC group. Scale bar represents 25 µm. **D** Bar plots showing the expression of *Cxcl10*, *Cybb*, and *Icam1 *in vitro neutrophils co-cultured with or without MSCs (*n* = 3). **E** Relative ROS levels were measured by DCFH-DA probe in lung neutrophil after co-cultured with or without MSCs. **F** Bar plot showing MFI of DCFH-DA probe in lung neutrophil after co-cultured with or without MSCs (*n* = 3). **G** Representative flow cytometric dot plots showing subpopulations of lung neutrophils defined by CD24 co-cultured with or without MSCs (*n* = 3). **H** Bar plot showing the proportion of CD24^hi^ neutrophils cultured in vitro with or without MSCs (*n* = 3). ns, no significance; *, *P* < 0.05; **, *P* < 0.01; ***, *P* < 0.001 unpaired Student’s *t* test

The aged neutrophils (Cluster 3) expressed a higher level of *Cd24a*, and the number of cells in Cluster 3 was higher on day 3 in MSC group. Based on this, we examined whether the frequency of neutrophils expressing CD24 differed after MSC treatment. As expected, the proportion of CD24^hi^ neutrophils was greatly increased in MSC group (Fig. 7A, B). Immunofluorescence staining also suggested that the expression of CD24 was increased after MSC therapy (Fig. 7C). Moreover, MSCs also inhibited the expression of pro-inflammatory factor genes when co-cultured with inflammatory neutrophils in vitro. The expression of chemokine gene *Cxcl10*, ROS-related gene *Cybb* (NOX2), and cell adhesion-related gene *Icam1* was significantly decreased after 2 h of co-culture with MSCs (Fig. 7D). The ROS levels were also downregulated by MSCs (Fig. 7E, F). Consistent with in vivo observations, the number of CD24^hi^ neutrophils was increased after co-culture with MSCs (Fig. 7G, H). The expressions of *Cxcl10*, *Cybb* and *Icam1* were downregulated and the ROS productions were decreased after cultured with MSCs using transwell assay (Additional file 1: Fig. S5). These findings indicated that MSC treatment shifted the pro-inflammatory phenotype of neutrophils by upregulating the expression of CD24 after 3 days.

MSC treatment reduced the number of activated neutrophils (Cluster 1), which had higher levels of specific, gelatinase, and secretory granules and increased chemotaxis, ROS production, and NADPH oxidase. At the same time, the number of aged neutrophils (Cluster 3), which exhibited reduced synthesis of granular proteins, chemotaxis, ROS production, and NADPH oxidase, was increased.

### Neutrophil functional changes on day 7 after MSC treatment

As mentioned earlier, in terms of neutrophil cluster distribution, LPS group and MSC group were the most similar on day 7. However, the neutrophil phenotypes changed significantly between days 3 and 7 after LPS instillation, with aged neutrophils (Cluster 2) becoming the predominant phenotype on day 7 as shown in Additional file 1: Fig S6a. The number of DEGs on day 7 in LPS group and MSC group was minimal (Additional file 1: Fig. S6b, Table S4). To confirm whether neutrophil functionality had been changed on day 7 after MSC treatment, GSEA analysis was performed. The GSEA of KEGG pathways showed that phagosomes were activated in neutrophils on day 7 after MSC treatment (Additional file 1: Fig. S6c). GO biological processes, proteasomal ubiquitin-independent protein catabolic processes, and positive regulation of cell killing were activated in MSC group on day 7 (Additional file 1: Fig. S6d). On day 7, inflammation in the lungs was reduced to normal levels and the residual neutrophils in MSC group might have engulfed tissue cell fragments and broke down proteins, thereby helping to restore the damaged lung tissue.

## Discussion

As reported, 15–30% hospitalized COVID-19 patients develop ARDS, and COVID‐19 ARDS is prone to worse outcomes than ARDS from other causes [35]. There is a 65.7–94% mortality rate in patients who received mechanical ventilation [36]. Accumulating preclinical researches [37, 38] and clinical trials [17, 19, 39] have shown that MSC therapy is a comprehensive treatment for ALI, and researches in the past 2 years have shown that MSC therapy can also improve the outcomes of patients with severe or critical COVID-19 [17–19]. Many indicators, such as the median time of hospital stay and symptoms remission, are shortened after MSC therapy. MSCs also modulate the immune system of patients with severe COVID-19. For example, the levels of C-reactive protein (CRP) and plasma pro-inflammatory cytokines are markedly decreased [17]. It has been reported that the pathological severity of ARDS induced by LPS is similar to that induced by COVID-19 [40]. Our study profiles neutrophils involved in LPS-induced lung injury and provide a novel mechanism by which MSCs can shift the pro-inflammatory phenotype of neutrophils by increasing CD24 expression.

First, we show that MSCs separated from C57BL/6 mice compact bone have therapeutic effects in LPS-induced ALI. IFN-γ and IL-6 are critical pro-inflammatory cytokines involved in acute inflammation. Researchers have identified a group of neutrophils expressing interferon-stimulated genes which are expanded during bacterial infection and gradually develop into aged neutrophils [33]. Plasma IL‐6 is a potential prognosis factors in COVID-19 severe patients and a vital factors between endothelial cells and neutrophils [41]. CXCL1 recruits neutrophils into lung tissue [42]. After MSC treatment, we observe not only restored lung tissue structure, but also decreased levels of IFN-γ, IL-6 and CXCL1 in BALF during the initial inflammatory phase.

After quality control, we reduce the data dimensionality and used tSNE algorithm to cluster cells. The genes associated with inflammation are in the top 20 genes in all dimensions, and nine different types of neutrophils are found. Because the proportions of Clusters 6–8 are minimal, and they exhibit high expression of *Ighm*, *Cd79a,* and *Ms4a4b*, which are likely expressed by B cells, these clusters are excluded from subsequent analyses.

*Cxcr2*, *Sell*, *Mmp8*, and *Mmp9* are highly expressed in circulatory neutrophils (Cluster 5) in healthy tissue (PBS group). CXCR2^hi^ neutrophils are mature neutrophils with high expression of gelatinase granule proteins [43], and MMP8 and MMP9 modulate epithelial and endothelial integrity to promote neutrophil transendothelial migration [44]. This suggests that neutrophils in healthy lung tissue are mature and act as supervisors to maintain immune homeostasis.

In the initial inflammatory phase (day 3 after LPS treatment), the heterogeneity of neutrophils is altered. There are three main clusters, two of which are activated neutrophils with high expression of *Cxcl10* and granule genes; the other one consists of aged neutrophils. Surprisingly, the therapeutic effects of MSCs on day 3 are mainly attributed the activated neutrophils (Cluster 1) and aged neutrophils (Cluster 3).

Previous research has shown that effector functions, including phagocytosis and ROS production, are enhanced in neutrophils overexpressing ICAM-1 [45]. During sepsis, ICAM-1^+^ neutrophils increase in number and accumulate in lung tissue, leading to ARDS [46]. Blockade of Cybb (NOX2) can ameliorate LPS-induced inflammation [47]. It has been reported that CD24 inhibits the inflammatory response to injury [48], and Parlato and colleagues show that CD24 can significantly trigger neutrophil apoptosis [49]. Moreover, CD24 has been shown to be downregulated in the neutrophils of sepsis patients. A decreased CD24-mediated cytotoxic response and neutrophil death are also associated with downregulated expression of CD24. Wang et al*.* report that a delay in human neutrophil apoptosis can be caused by increased PD-L1 expression, leading to lung injury and increased mortality during sepsis [50]. IFN-γ can induce PD-L1 expression in neutrophils [51], and interferon-stimulated genes (ISGs) are important in inflammatory neutrophils. Deng and colleagues report that neutrophils in lupus patients exhibit higher ISG activity [52], and the number of ISG-related neutrophils is increased in both humans and mice [33].

In the current study, the activated neutrophils (Cluster 1) express higher levels of *Icam1*, *Cd24a*, *Cd274,* and *Isg15*. Genes that are significantly downregulated by MSCs are the marker genes for Cluster 1, and the frequency of Cluster 1 cells is reduced after MSC treatment. MSCs have also been shown to reduce the expression of *Cxcl10*, *Cybb,* and *Icam1 *in vitro. These mechanisms may explain why, in our study, MSC treatment inhibits the inflammatory neutrophil phenotype in the initial inflammatory phase.

On the other hand, some of the genes significantly downregulated by MSCs are marker genes for Cluster 3, and an increase in the frequency of Cluster 3 neutrophils is observed in the initial inflammatory phase. Cluster 3 is displayed increased expression of CD24, and it has been reported in previous studies that CD24 has a negative effect on neutrophils [49]. In our flow cytometry data, we observe an increase in the number of CD24^hi^ neutrophils after MSC treatment, and the expression of CD24 on inflammatory neutrophils is upregulated after MSC co-culture. These results suggest that the inflammatory neutrophil phenotype is shifted toward increased expression of CD24, which may be responsible for the therapeutic effects of MSC treatment in the initial inflammatory phase of LPS-induced ALI. In addition, the therapeutic effect of MSC treatment also includes a reduction in chemotaxis, ROS production, and NADPH oxidase, as well as the inhibition of specific and gelatinase granules, and secretory vesicles.

The types of neutrophils differ minimally on day 7 after MSC treatment, but their functions are different. Phagosomes, proteasomal ubiquitin-independent protein catabolic processes, and positive regulation of cell killing are activated in neutrophils after MSC treatment. The number of Cluster 2 neutrophils that highly expressed *Cxcr4* increases significantly on day 7 after LPS treatment. CXCR4 may help clear aged neutrophils and impede neutrophil homing to the bone marrow [32, 53]. Xie et al. [33] describe that neutrophils with high expression of CXCR4 have an aged phenotype. Therefore, neutrophils exhibiting an aged phenotype and high *Cxcr4* expression in the recovery phase of ALI may induce neutrophils to exit lung tissue to find “invaders” in other organs.

Regrettably, we have no the clinical patients to further study the functions of neutrophils in ALI after MSC treatment. Furthermore, basic experiments are needed to validate our neutrophil scRNA-Seq clustering data and identify which molecules enable MSCs to shift the pro-inflammatory phenotype of neutrophils (by upregulating the expression of CD24).

## Conclusions

In conclusion, our data show that MSCs can ameliorate LPS-induced ALI. The neutrophils infiltrating into lung tissue are heterogeneous and can be divided into circulatory, activated, and aged neutrophils. MSC treatment shifts the activated neutrophil phenotype into an aged neutrophil phenotype by upregulating the expression of CD24, thereby reducing chemotaxis, ROS production, NADPH oxidation, and the secretion of granules, which ultimately facilitate the recovery of ALI. Our results provide new insight into the therapeutic mechanism of MSC treatment in ALI.

### Supplementary Information

**Additional file 1:** Supplemetary methods and **Figure S1.** Characteristics of MSCs; **Figure S2.** Quality control of single-cell RNA sequencing data; **Figure S3.** Heatmaps showing the top 20 genes of the 15 PCs; **Figure S4.** The differences between cluster 1 and 3; **Figure S5.** MSCs could inhibit neutrophils function through paracrine effects; **Figure S6.** The changes of lung neutrophil function between LPS group and MSC group on day 7; **Figure S7.** Results of western blot analysis showed that the levels of NOX2 protein were decreased after MSC treatment; **Table S1.** Top 10 marker genes of each cluster; **Table S2.** Functional signatures with gene list; **Table S3.** Results of differential gene expression analysis between LPS group and MSC group on day 3; **Table S4.** Results of differential gene expression analysis between LPS group and MSC group on day 7.

## Data Availability

We submitted the raw sequence data to the Genome Sequence Archive of the Beijing Institute of Genomics Data Center, Chinese Academy of Sciences (accession number CRA003097), and publicly available at https://bigd.big.ac.cn/gsa.

## Abbreviations

ALI: Acute lung injury
ARDS: Acute respiratory distress syndrome
BALF: Bronchoalveolar lavage fluid
BSA: Bovine serum albumin
CRP: C-reactive protein
DC: Dendritic cell
DCFH-DA: 2′,7′-Dichlorodihydrofluorescein diacetate
DEGs: Differentially expressed genes
FBS: Fetal bovine serum
GO: Gene ontology
GSEA: Gene set enrichment analysis
HE: Hematoxylin and eosin
ICUs: Intensive care units
IFN-γ: Interferon-γ
IL-6: Interleukin-6
IMDM: Iscove’s modified Dulbecco’s medium
IGF1: Insulin-like growth factor I
ISGs: Interferon-stimulated genes
KEGG: Kyoto Encyclopedia of Genes and Genomes
LPS: Lipopolysaccharide
MSC: Mesenchymal stem cell
NADPH: Nicotinamide adenine dinucleotide phosphate
PBS: Phosphate-buffered saline
PCA: Principal component analysis
PGE2: Prostaglandin E2
ROS: Reactive oxygen species
scRNA sequencing: Single-cell RNA sequencing
UMI: Unique molecular identifier
