# Supplementary material for: Mesenchymal stem cells shift the pro-inflammatory phenotype of neutrophils to ameliorate acute lung injury
Source: Stem Cell Res Ther. 2023 Aug 8;14:197. doi: 10.1186/s13287-023-03438-w (PMC10408228; doi:10.1186/s13287-023-03438-w)
Supplement: Supplementary file 1 — Additional file 1: Supplemetary methods and Figure S1. Characteristics of MSCs; Figure S2. Quality control of single-cell RNA sequencing data; Figure S3. Heatmaps showing the top 20 genes of the 15 PCs; Figure S4. The differences between cluster 1 and 3; Figure S5. MSCs could inhibit neutrophils function through paracrine effects; Figure S6. The changes of lung neutrophil function between LPS group and MSC group on day 7; Figure S7. Results of western blot analysis showed that the levels of NOX2 protein were decreased after MSC treatment; Table S1. Top 10 marker genes of each cluster; Table S2. Functional signatures with gene list; Table S3. Results of differential gene expression analysis between LPS group and MSC group on day 3; Table S4. Results of differential gene expression analysis between LPS group and MSC group on day 7. [file 13287_2023_3438_MOESM1_ESM.docx]

## **Supplementary information**

Mesenchymal stem cells shift the proinflammatory phenotype of neutrophils to ameliorate acute lung injury

Bing Feng^1,3^, Xudong Feng^1,3^, Yingduo Yu^1,3^, Haoying Xu^1,3^, Qingqing Ye^1,3,4^, Ruitian Hu^5^, Xinru Fang^1,3^, Feiqiong Gao^1,3^, Jian Wu^1,3^, Qiaoling Pan^1,3^, Jiong Yu^1,3^, Guanjing Lang^1,3^, Lanjuan Li^1,2,3^, Hongcui Cao^*1,3,4^

1 State Key Laboratory for the Diagnosis and Treatment of Infectious Diseases, The First Affiliated Hospital, Zhejiang University School of Medicine, 79 Qingchun Rd., Hangzhou City 310003, China

2 Jinan Microecological Biomedicine Shandong Laboratory, Jinan, Shandong 250117, China

3 National Clinical Research Center for Infectious Diseases, 79 Qingchun Rd., Hangzhou City 310003, China

4 Key Laboratory of Diagnosis and Treatment of Aging and Physic-chemical Injury Diseases of Zhejiang Province, 79 Qingchun Rd, Hangzhou City 310003, China

5 Department of Chemistry, Duke University, 124 Science Drive, Durham, NC 27708, United States

## **Supplementary Methods**

**Isolation, purification and differentiation of MSCs**

MSCs were isolated and cultured according to Zhu *et al* [1]. Humeri, tibiae and femurs were obtained from 1-2 week mice, cut into pieces, and manipulated by 2 mg/mL collagenase II digestion solution (Gibco Life Technologies, Grand Island, NY, USA) for 1.5 h at 37°C. Tissue pieces were cultured in 7.5 mL C57BL/6-MSC special complete MEM (OriCell™ C57BL/6 MSC Complete Medium; Cyagen Biosciences, Guangzhou, China). After 3 days, nonadherent cells were removed. After 5 days, the cells were passaged with trypsin (0.25%)–EDTA, and MSCs in passage 3 were used for experiments. The osteoinductive medium (OriCell™ C57BL6 MSC Osteogenic Differentiation Medium; Cyagen Biosciences) and the adipogenic culture medium (OriCell™ C57BL6 MSC Adipogenic Differentiation Medium; Cyagen Biosciences) were used for inducing osteogenic and adipogenic differentiation. Alizarin Red S (Cyagen Biosciences) staining and Oil red O (Cyagen Biosciences) staining were used to identify results of differentiation.

## **Animal models of acute lung injury and MSCs treatment**

6–8 weeks old, specific pathogen-free C57BL/6 male mice were purchased from Nanjing Biomedical Research Institute of Nanjing University. All mice were housed in the Experimental Animal Center of Zhejiang University. Lipopolysaccharide (LPS, Sigma-Aldrich, MO, USA) was dissolved in phosphate buffer saline (PBS). The solution was given intratracheally to mice at a concentration of 20 μg LPS depending on mice weight. After 4 hours, 20 μL PBS containing 2% mouse serum with or without 5 × 10^5^ compact bone derived mesenchymal stem cells (MSCs) were given intratracheally, respectively named as LPS/MSC group (MSC group) and LPS/PBS group (LPS group). As the vehicle control group (PBS group), PBS was given intratracheally firstly, and 20 μL of PBS containing 2% mouse serum was given after 4 hours. There were 5, 10 and 10 mice in PBS group, LPS group, and MSC group, respectively. The mice were sacrificed on 3 and 7 days after MSCs or PBS administration to obtain the lung tissues for following experiments.

## **Lung Histology**

Mice were sacrificed and isolated lungs were fixed using 4% paraformaldehyde. After fixation for one day, the lungs were embedded in paraffin, and cut into 5 µm thick sections. The lung sections were stained with hematoxylin and eosin (H&E). The lung slides were scanned by NanoZoomer-SQ (Hamamatsu Corp., Hamamatsu, Japan) and the typical images were exported by NDP.view.2 software (Hamamatsu Corp.) at a magnification of ×20.

**Single-cell RNA sequencing data processing**

***Analysis of differentially expressed genes***

We used the FindAllMarker function (test.use = “wilcox”, min.pct = 0.1, logfc.threshold = log [1.2]) to obtain the marker genes for each cluster. The top 10 marker genes were filtered by “avg_logFC”, and arranged from high to low with a *P* value < 0.05. Supplementary Table S1 lists the top 10 marker genes for each cluster. The differentially expressed genes (DEGs) in each sample were analyzed with the Findmarker function (test.use = “wilcox”, logfc.threshold = log [1.2]) and DEGs with *P* value < 0.05 were selected. Gene Ontology (GO) and Kyoto Encyclopedia of Genes and Genomes (KEGG) enrichment analyses, and gene set enrichment analysis (GSEA) analysis, were performed with the R package “clusterProfiler” [2]. *P* value < 0.05 were considered statistically significant.

***Scoring of biological processes***

Functional scores were defined by the AddModuleScore function (ctrl = 5) using the full gene list from Xie et al [3]. We list all of the genes used in this research in Supplementary Table S2. According to Xie et al.[3], aged neutrophils have reduced expression of *Sell*, *Cxcr2* and *Cd47*, and increased expression of *Itgam*, *Itga4*, *Itgax*, *Tlr4*, *Icam1*, *Cxcr4*, and *Cd24a*. Therefore, the negatively scaled expression of *Sell*, *Cxcr2,* and *Cd47*, and the scaled expression of *Itgam*, *Itga4*, *Itgax*, *Tlr4*, *Icam1*, *Cxcr4*, and *Cd24*, were summed to obtain the neutrophil score.

***Analysis of Pseudotime trajectory***

The R package Monocle (ver. 2, Bioconductor) was used to predict the potential lineage differentiation trajectory. The Seurat object was transformed into the CellDataSet object using the as.CellDataSet function. The trajectory was ordered based on genes with qval < 0.01 after applying the estimateSizeFactors and estimateDispersions functions.

**Flow cytometry**

Antibodies used for the immune cells separated from lung tissues and separated neutrophils included PE anti-mouse Ly6G (1A8, BioLegend), BV421 anti-mouse CD24 (M1-69, BioLegend) and FVS 780 (BD Biosciences). Antibodies used for characteristics of MSCs included APC anti-mouse CD29 (HMβ1-1, Biolegend), APC-Cy7 anti-mouse CD11b (M1/70; Biolegend), APC-Cy7 anti-mouse MCHI-1A (M5/114.15.2; Biolegend), PE anti-mouse CD44 (IM7; Biolegend), PE anti-mouse SCA-1 (D7; Biolegend), PE-Cy7 anti-mouse CD31 (390; Biolegend), PerCP anti-mouse CD86 (GL-1; Biolegend), and PE-Cy7 anti-mouse CD45 (30-F11; Biolegend). The flow cytometry was performed using CytoFLEX LX (Beckman Coulter, CA, USA).The data were analyzed by FlowJo software (Tree Star, OR, USA). For gating strategy, once the doublets (by FSC-H vs. FSC-A) and dead cells were excluded, neutrophils were identified as Ly6G^+^, and the expression levels of CD24 were analysis between LPS group and MSC group (data not shown).

**Immunofluorescence staining**

The paraffin sections were placed in 65°C oven for 1 h. After deparaffin, distilled water was used to wash the slides. Citrate (G1201, Servicebio, Wuhan, China) was used for heat mediated antigen retrieval. After blocking endogenous peroxidase, the sheep serum was used for blocking. The slides were incubated with Ly6G antibody (ab25377, Abcam) and CD24 antibody (ab290730, Abcam) overnight at 4°C. After washing, donkey anti‐rat IgG (Alexa Fluor™ 594, A-21209, Thermo Fisher, Rockford, IL, USA) and goat anti‐rabbit IgG (Alexa Fluor® 647, ab150083, Abcam) were used for the secondary antibody. The nuclei were stained with DAPI. The sections were photographed using a confocal laser scanning microscope (Zeiss LSM710; Carl Zeiss AG, Germany).

**RT-qPCR**

The neutrophils cultured in vitro were collected gently and washed with PBS. Total RNA was harvested using EZ-press RNA Purification Kit according to the manufacturer’s instructions (EZBioscience, MN, USA). The cDNA was subsequently synthesized using a Reverse Transcription Kit (Qiagen, Hilden, Germany) and qPCR was performed by SYBR Premix Ex Taq™ II Kit (Takara Bio Inc., Shiga, Japan). The primers were as follows: Cxcl10: forward 5’-CCCACGTGTTGAGATCATTG-3’ and reverse 5’-GCTCTCTGCTGTCCATCCAT-3’; Cybb: forward 5’-CTTGGAAATGGATAGTGGGTCCT-3’ and reverse 5’-TGTACCAGACAGACTTGAGAATGGA-3’; and Icam1: forward 5’-GTGATGCTCAGGTATCCATCCA-3’ and reverse 5’-CACAGTTCTCAAAGCACAGCG-3’.

**Western blot analysis**

Lung neutrophils separated from LPS group and MSC group day3 were solubilized in RIPA lysis buffer (89900, Thermo Fisher, Rockford, IL, USA) with 1 × phosphatase inhibitors (78420, Thermo Fisher, Rockford, IL, USA) and protease inhibitor (87786, Thermo Fisher, Rockford, IL, USA). The protein concentrations were detected using a BCA kit (Beyotime Biotechnology, Shanghai, China). The western blot was conducted as previously described [4]. Briefly, proteins were run on SDS-PAGE gels (GenScript, Nanjing, China) and transferred to polwvinylidene fluoride membranes (Merck-Millipore, Darmstadt, Germany). After being blocked in QuickBlock™ Blocking Buffer (Beyotime Biotechnology, Shanghai, China), the membranes were immunoblotted with NOX2 (SC-130543; Santa Cruz) and GAPDH (D16H11, Cell Signaling Technology) overnight at 4°C. Tris-buffered saline with 0.1% Tween was used to wash membranes 3 times. Goat anti-rabbit IgG H&L (HRP) (ab6721; Abcam) and rabbit anti-mouse IgG H&L (HRP) (ab6728; Abcam) were used for secondary antibody. After washing, Pierce™ ECL Western blot analysis Substrate (Thermo Fisher, Rockford, IL, USA) was used to incubate the membranes, and the ChemiScope Western Blot Imaging System (Clinx Science Instruments Co., Ltd., Shanghai, China) was used to capture the image.

**References**

1. Zhu H, Guo ZK, Jiang XX, Li H, Wang XY, Yao HY, et al. A protocol for isolation and culture of mesenchymal stem cells from mouse compact bone. Nat Protoc. 2010; 5(3):550-60.

2. Yu G, Wang L-G, Han Y and He Q-Y. clusterProfiler: an R package for comparing biological themes among gene clusters. OMICS. 2012; 16(5):284-87.

3. Xie X, Shi Q, Wu P, Zhang X, Kambara H, Su J, et al. Single-cell transcriptome profiling reveals neutrophil heterogeneity in homeostasis and infection. Nat Immunol. 2020; 21(9):1119-33.

4. Feng X-D, Zhou J-H, Chen J-Y, Feng B, Hu R-T, Wu J, et al. Long non-coding RNA SNHG16 promotes human placenta-derived mesenchymal stem cell proliferation capacity through the PI3K/AKT pathway under hypoxia. World J Stem Cells. 2022; 14(9):714-28.

## **Supplementary figures and figure legends**


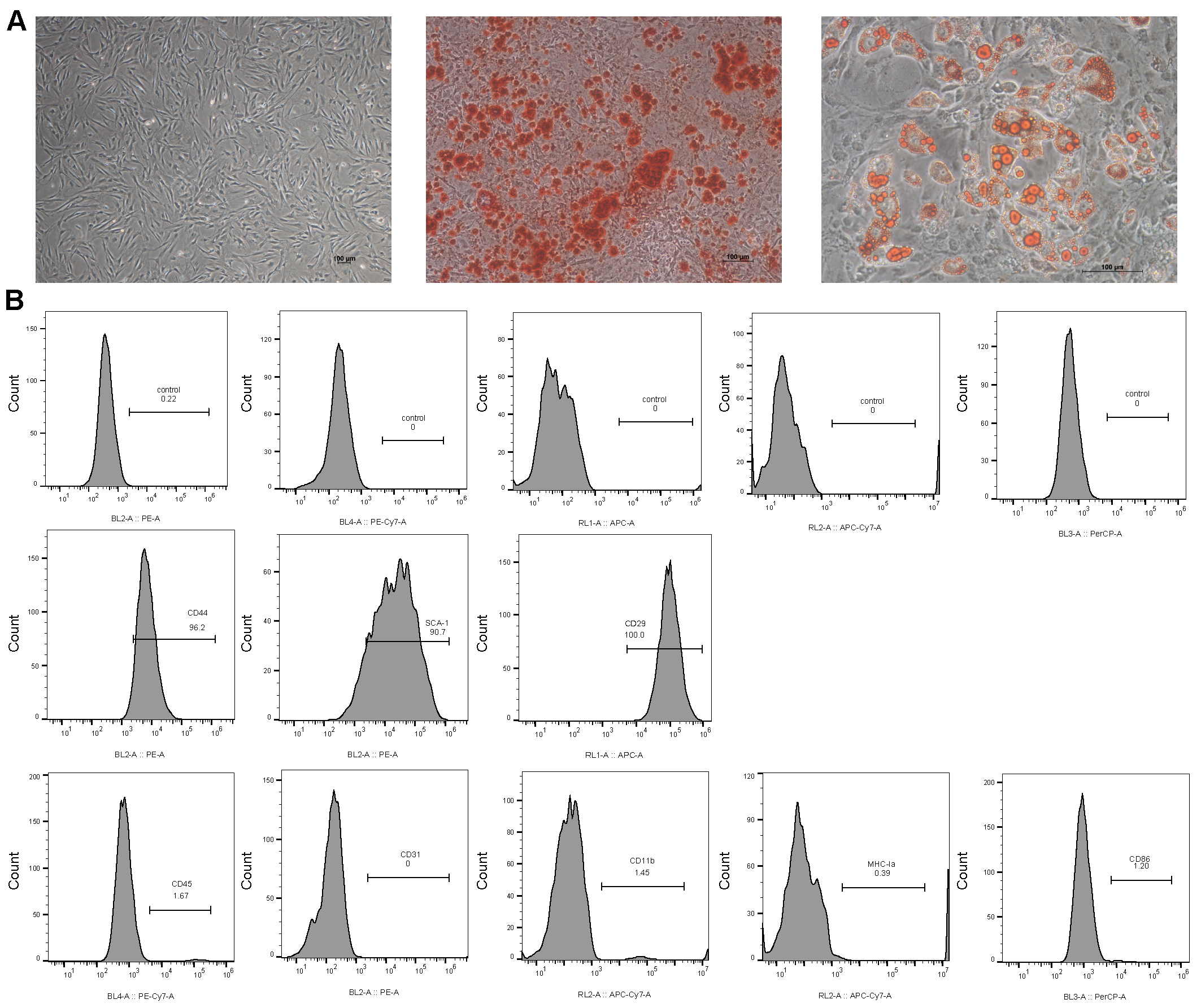


**Supplementary Figure S1. Characteristics of MSCs.**

**A** MSCs were spindle-shaped in passage 3. MSCs differentiated into osteocytes and adipocytes. **B** FACS analysis of MSCs using monoclonal antibodies including CD44, SCA-1, CD29, CD45, CD31, CD11b, MHC-Ia, and CD86.


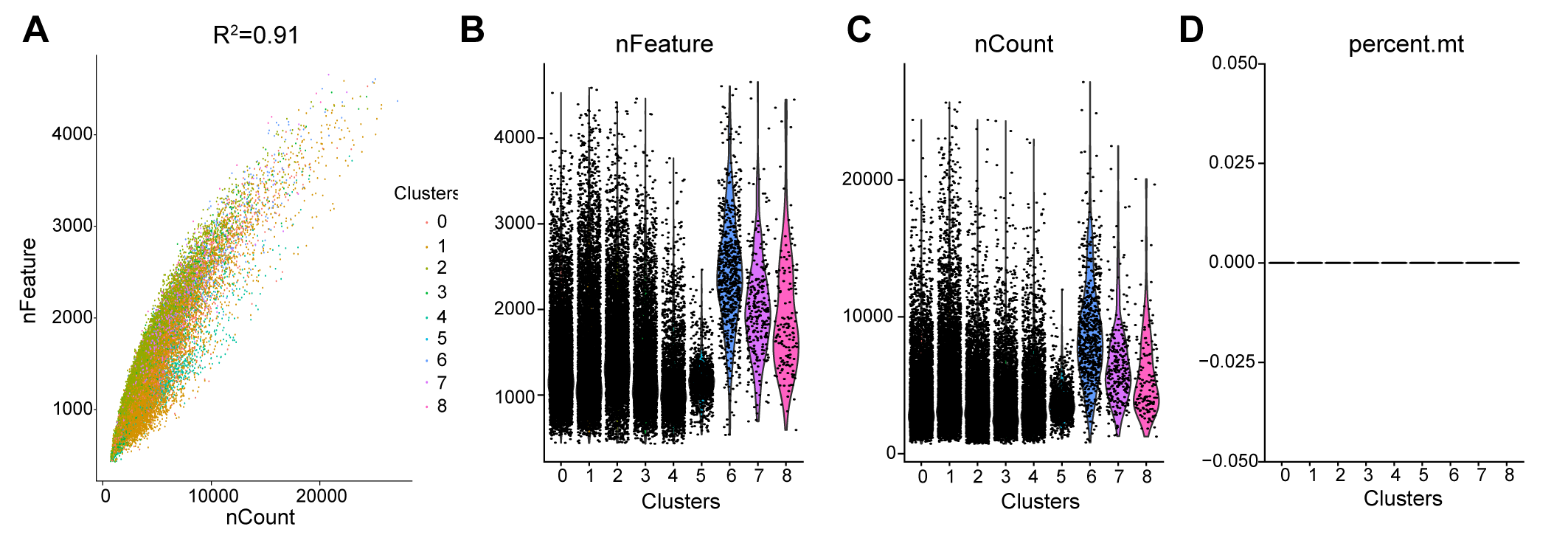


**Supplementary Figure S2. Quality control of single-cell RNA sequencing data.**

**A** Dot plot of nCount and nFeature. **B** Plots depicting the number of genes detected in each cell (nFeature). **C** Plots depicting the number of unique RNA molecules detected in each cells (nCount). **D** Plots depicting the percentage of reads mapping to the mitochondrial genome (percent.mt).


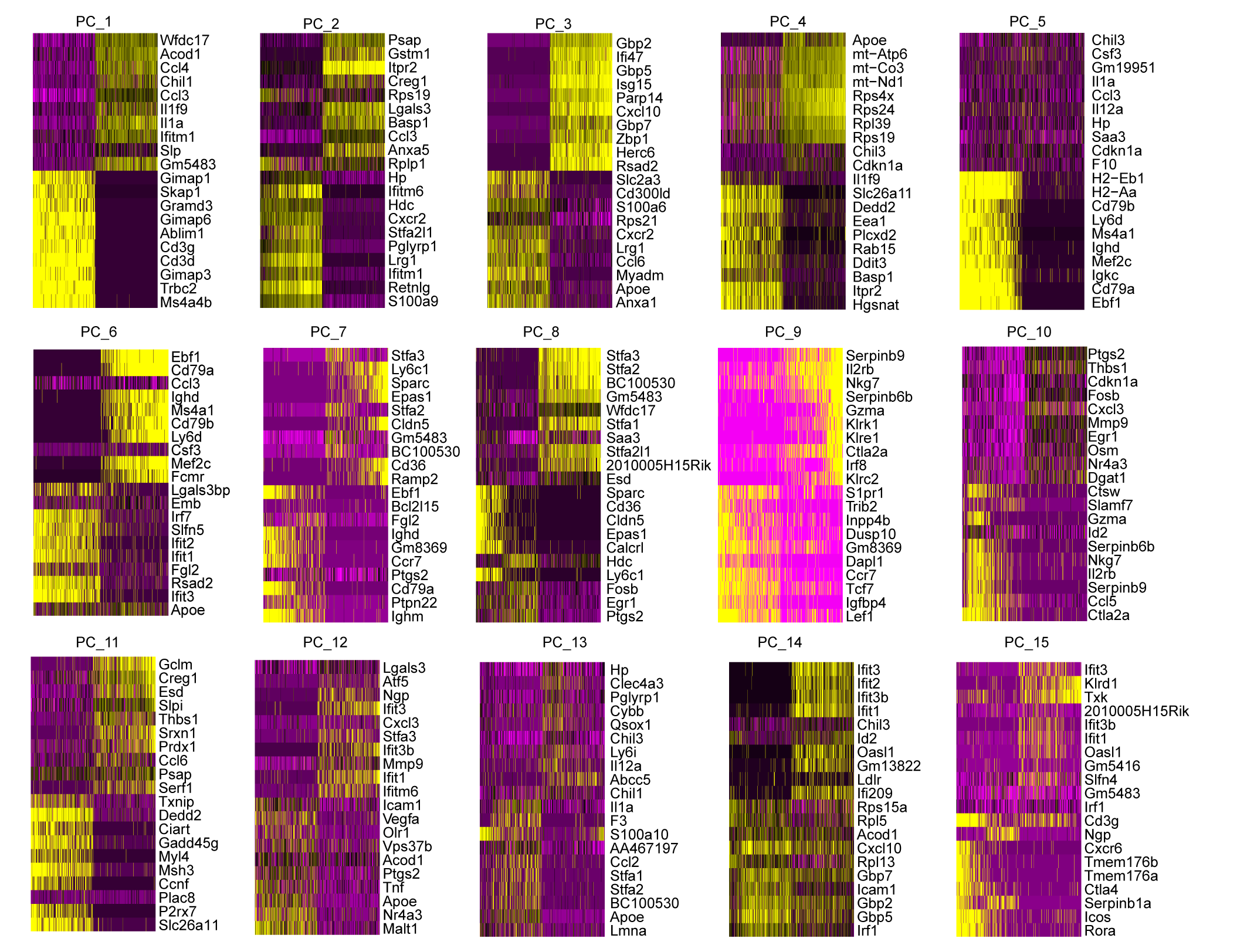


**Supplementary Figure S3. Heatmaps showing the top 20 genes of the 15 PCs.**


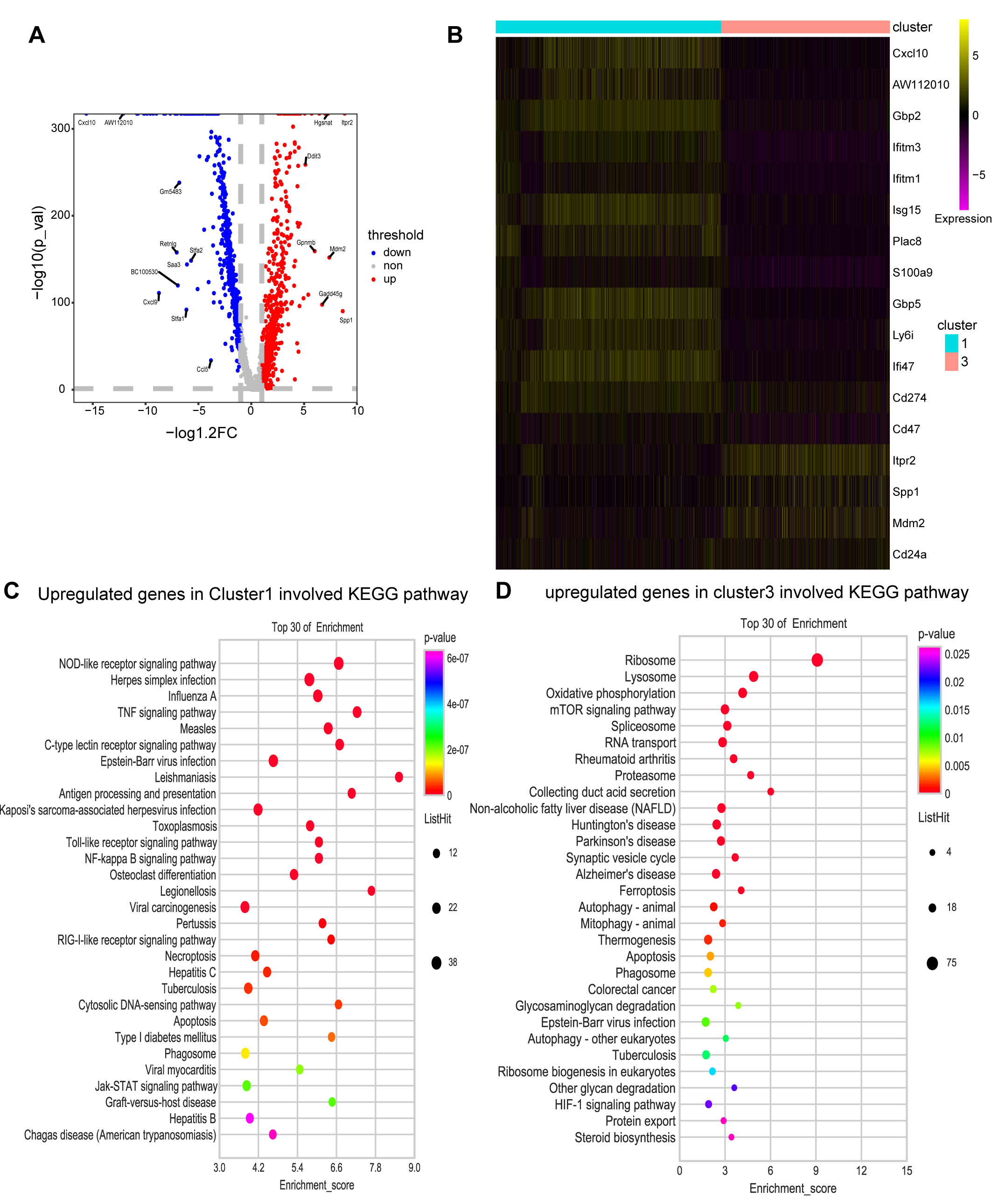


**Supplementary Figure S4. The differences between cluster 1 and 3.**

**A** Volcano plot showing the DEGs of cluster 3 and cluster 1. *P* value was calculated using Wilcoxon test. The genes with *P* value < 0.05 and foldchange > 1.2 were selected. Blue dots represented the downregulated genes in cluster 3, and red dots represented the upregulated genes in cluster 3. **B** Heatmap showing the expression of selected genes between cluster 1 and 3. **C** The bubble chart showing top 30 functional KEGG enrichment analysis using the upregulated DEGs in cluster 1 with foldchange >1.2 and *P* value < 0.05. **D** The bubble chart showing top 30 functional KEGG enrichment analysis using the upregulated DEGs with foldchange >1.2 and *P* value < 0.05 in cluster 3.

**
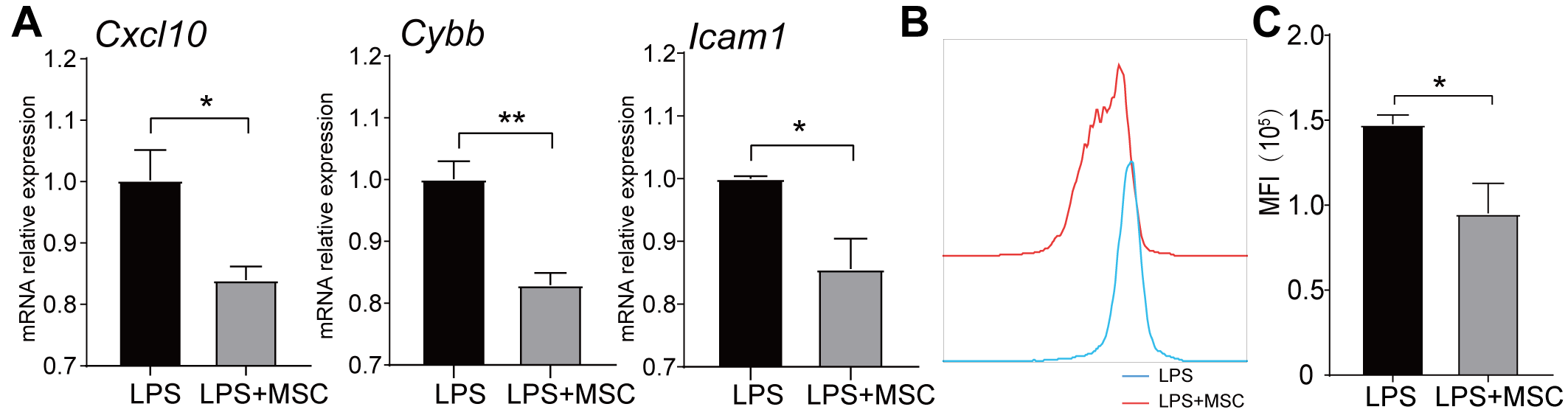
**

**Supplementary Figure S5. MSCs could inhibit neutrophil functions through paracrine effects**

**A** Bar plots showing the expressions of *Cxcl10*, *Cybb*, and *Icam1* in neutrophils cultured with or without MSCs through transwell assay (n = 3). **B** Relative ROS levels were measured by DCFH-DA probe in lung neutrophil after cultured with or without MSCs using transwell assay. **C** Bar plot showing MFI of DCFH-DA probe in lung neutrophil after cultured with or without MSCs using transwell assay (n = 3). *, *P* < 0.05; **, *P* < 0.01; unpaired Student’s t test.


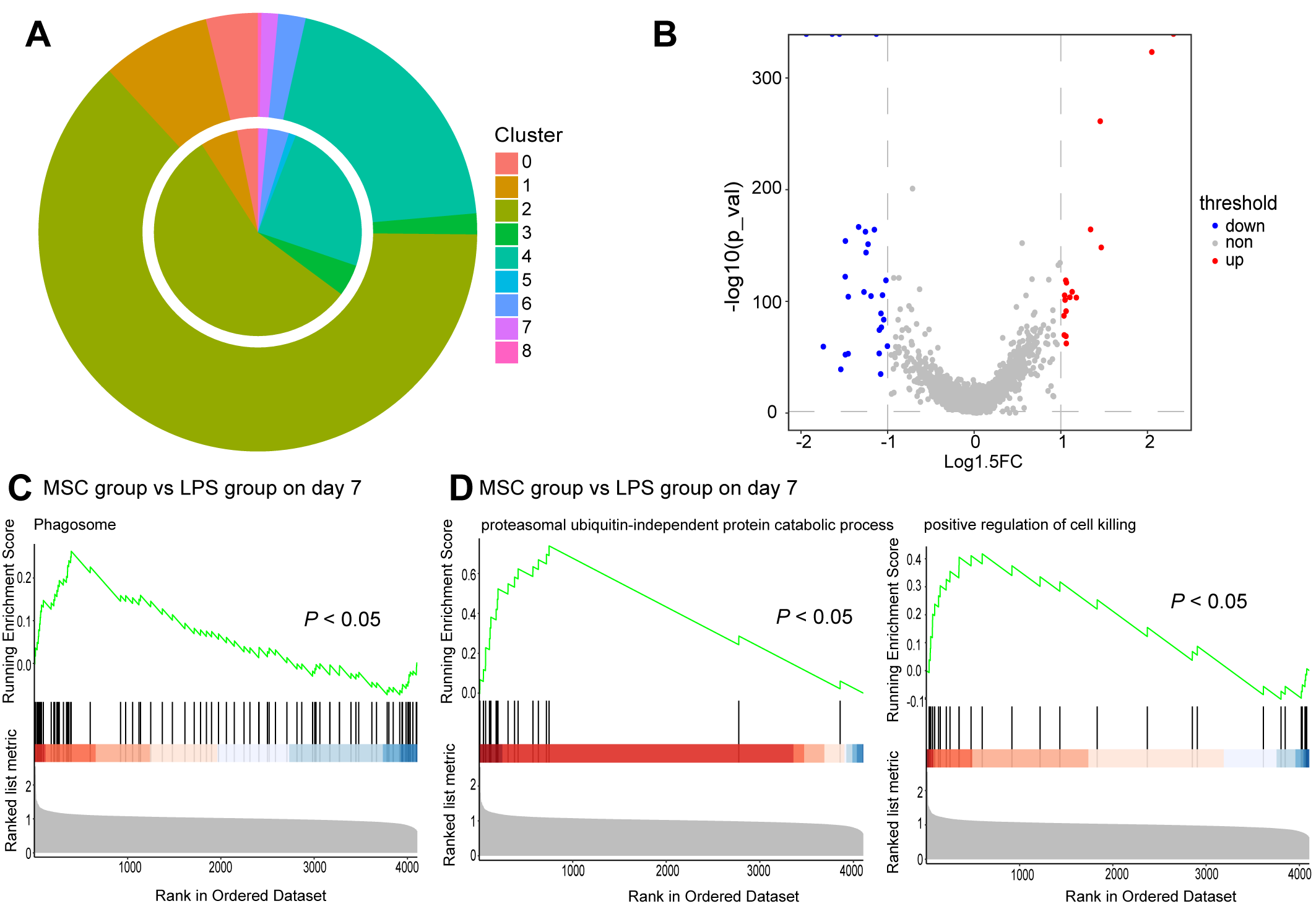


**Supplementary Figure S6. The changes of lung neutrophil function between** **LPS group and MSC group on day 7.**

**A** The distributions of 9 clusters in LPS group and MSC group on day 7. The inner ring represented LPS group on day 7 and the outer ring represented MSC group on day 7. **B** Volcano plot showing the DEGs of LPS group and MSC group on day 7. *P* value was calculated using Wilcoxon test. The genes with *P* value < 0.05 and foldchange > 1.2 were selected. Blue dots represented the genes downregulated by MSCs , and red dots represent the genes upregulated by MSCs on day 7. (**C, D**) Gene-set enrichment analysis (GSEA), showing that the DEGs between LPS group and MSC group on day 7 were significantly enriched in Phagosome (mmu04145), proteasomal ubiquitin-independent protein catabolic process (GO: 0010499) and positive regulation of cell killing (GO: 0031343).


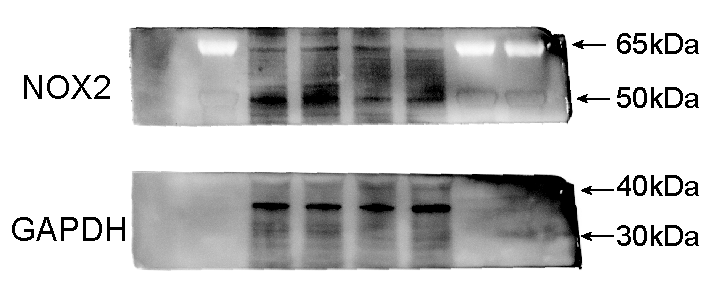


**Supplementary Figure S7. Results of western blot analysis showed that the levels of NOX2 protein were decreased after MSC treatment.**

## **Supplementary tables**

**Table S1.** **Top 10 marker genes of each cluster.**

| Gene | p_val | avg_logFC | pct.1 | pct.2 | p_val_adj | cluster |
| --- | --- | --- | --- | --- | --- | --- |
| Chil3 | 0 | 0.824749831 | 0.931 | 0.766 | 0 | 0 |
| Gm19951 | 0 | 0.752382768 | 0.567 | 0.305 | 0 | 0 |
| Wfdc21 | 0 | 0.732349156 | 0.967 | 0.878 | 0 | 0 |
| Fpr1 | 0 | 0.65970343 | 0.814 | 0.558 | 0 | 0 |
| Lcn2 | 0 | 0.622005801 | 0.989 | 0.933 | 0 | 0 |
| Irak3 | 0 | 0.612150765 | 0.842 | 0.574 | 0 | 0 |
| Pou2f2 | 0 | 0.58600467 | 0.597 | 0.337 | 0 | 0 |
| Ltb | 0 | 0.522876322 | 0.552 | 0.35 | 0 | 0 |
| Cxcl3 | 8.69E-263 | 0.901119153 | 0.428 | 0.249 | 2.70E-258 | 0 |
| Csf3 | 1.88E-200 | 0.519594268 | 0.476 | 0.304 | 5.83E-196 | 0 |
| Cxcl10 | 0 | 2.441765299 | 0.649 | 0.178 | 0 | 1 |
| Gbp2 | 0 | 1.825023051 | 0.964 | 0.36 | 0 | 1 |
| Gbp5 | 0 | 1.55872405 | 0.769 | 0.122 | 0 | 1 |
| Isg15 | 0 | 1.548291042 | 0.803 | 0.24 | 0 | 1 |
| Ifi47 | 0 | 1.501509246 | 0.788 | 0.157 | 0 | 1 |
| AW112010 | 0 | 1.411386307 | 0.601 | 0.314 | 0 | 1 |
| Rsad2 | 0 | 1.274812284 | 0.546 | 0.14 | 0 | 1 |
| Gbp7 | 0 | 1.168554574 | 0.804 | 0.262 | 0 | 1 |
| Parp14 | 0 | 1.161428455 | 0.807 | 0.281 | 0 | 1 |
| Cxcl9 | 4.89E-300 | 1.54802355 | 0.142 | 0.032 | 1.52E-295 | 1 |
| Apoe | 0 | 1.735632528 | 0.936 | 0.263 | 0 | 2 |
| mt-Nd1 | 0 | 1.118688267 | 0.808 | 0.48 | 0 | 2 |
| Hspa1b | 0 | 1.012438977 | 0.239 | 0.077 | 0 | 2 |
| Hexb | 0 | 0.856224013 | 0.674 | 0.339 | 0 | 2 |
| Uba52 | 0 | 0.834844438 | 0.732 | 0.373 | 0 | 2 |
| Klf4 | 0 | 0.800623952 | 0.367 | 0.143 | 0 | 2 |
| Lmna | 0 | 0.78734169 | 0.488 | 0.243 | 0 | 2 |
| mt-Atp6 | 0 | 0.783208172 | 0.985 | 0.907 | 0 | 2 |
| Nr4a2 | 0 | 0.781213778 | 0.246 | 0.07 | 0 | 2 |
| Vegfa | 0 | 0.775218497 | 0.364 | 0.163 | 0 | 2 |
| Itpr2 | 0 | 1.854097564 | 0.671 | 0.3 | 0 | 3 |
| Hgsnat | 0 | 1.514156532 | 0.542 | 0.156 | 0 | 3 |
| Mdm2 | 0 | 1.507196232 | 0.629 | 0.447 | 0 | 3 |
| Cstb | 0 | 1.373852307 | 0.995 | 0.842 | 0 | 3 |
| Gstm1 | 0 | 1.140546785 | 0.54 | 0.174 | 0 | 3 |
| Lgals3 | 0 | 1.132851877 | 0.913 | 0.764 | 0 | 3 |
| Basp1 | 0 | 1.112579383 | 0.853 | 0.43 | 0 | 3 |
| Inhba | 0 | 1.110274444 | 0.383 | 0.147 | 0 | 3 |
| Gadd45g | 3.20E-128 | 1.146556098 | 0.268 | 0.151 | 9.93E-124 | 3 |
| Spp1 | 6.66E-95 | 1.504232545 | 0.297 | 0.194 | 2.07E-90 | 3 |
| Retnlg | 0 | 2.975812282 | 0.965 | 0.297 | 0 | 4 |
| BC100530 | 0 | 2.755977439 | 0.564 | 0.124 | 0 | 4 |
| Stfa2 | 0 | 2.262469816 | 0.539 | 0.115 | 0 | 4 |
| Stfa1 | 0 | 2.119586574 | 0.449 | 0.109 | 0 | 4 |
| Ngp | 0 | 1.956815094 | 0.337 | 0.039 | 0 | 4 |
| Ifitm6 | 0 | 1.838634071 | 0.641 | 0.128 | 0 | 4 |
| S100a9 | 0 | 1.74823572 | 0.999 | 0.934 | 0 | 4 |
| Ifitm1 | 0 | 1.743080462 | 0.931 | 0.593 | 0 | 4 |
| Lrg1 | 0 | 1.72791419 | 0.851 | 0.186 | 0 | 4 |
| Stfa3 | 0 | 1.559027329 | 0.478 | 0.118 | 0 | 4 |
| G0s2 | 0 | 1.757663377 | 0.902 | 0.451 | 0 | 5 |
| Hdc | 0 | 1.747106344 | 0.993 | 0.538 | 0 | 5 |
| Cd300ld | 0 | 1.677456646 | 0.897 | 0.206 | 0 | 5 |
| Cxcr2 | 0 | 1.663572905 | 0.978 | 0.398 | 0 | 5 |
| Sorl1 | 0 | 1.624735578 | 0.967 | 0.41 | 0 | 5 |
| Slc2a3 | 0 | 1.574678216 | 0.673 | 0.11 | 0 | 5 |
| Fgl2 | 0 | 1.56762477 | 0.816 | 0.225 | 0 | 5 |
| Kctd12 | 0 | 1.535694758 | 0.864 | 0.295 | 0 | 5 |
| Csf3r | 0 | 1.493077805 | 0.996 | 0.681 | 0 | 5 |
| Lrg1 | 0 | 1.490904353 | 0.888 | 0.237 | 0 | 5 |
| Tmsb10 | 0 | 2.125931625 | 0.961 | 0.34 | 0 | 6 |
| Rpl12 | 0 | 1.812878017 | 0.975 | 0.668 | 0 | 6 |
| Trbc2 | 0 | 1.775046192 | 0.786 | 0.01 | 0 | 6 |
| Rpl3 | 0 | 1.738203077 | 0.948 | 0.455 | 0 | 6 |
| Ms4a4b | 0 | 1.68739755 | 0.805 | 0.006 | 0 | 6 |
| Rps4x | 0 | 1.66876093 | 0.977 | 0.635 | 0 | 6 |
| Rps24 | 0 | 1.660608095 | 0.994 | 0.893 | 0 | 6 |
| Rpl10a | 0 | 1.589458573 | 0.952 | 0.483 | 0 | 6 |
| Gzma | 1.12E-223 | 1.672491705 | 0.153 | 0.011 | 3.49E-219 | 6 |
| Ccl5 | 1.71E-127 | 2.205314326 | 0.488 | 0.168 | 5.32E-123 | 6 |
| Igkc | 0 | 3.057734043 | 0.859 | 0.054 | 0 | 7 |
| H2-Aa | 0 | 2.407912313 | 0.915 | 0.088 | 0 | 7 |
| H2-Eb1 | 0 | 2.200822267 | 0.9 | 0.088 | 0 | 7 |
| H2-Ab1 | 0 | 2.190076754 | 0.926 | 0.105 | 0 | 7 |
| Cd79a | 0 | 2.130009231 | 0.848 | 0.005 | 0 | 7 |
| Ighm | 0 | 2.027879004 | 0.907 | 0.071 | 0 | 7 |
| Ebf1 | 0 | 2.027763523 | 0.941 | 0.004 | 0 | 7 |
| Ly6d | 0 | 1.66943359 | 0.659 | 0.003 | 0 | 7 |
| Mef2c | 0 | 1.535439365 | 0.778 | 0.011 | 0 | 7 |
| Cd74 | 5.94E-203 | 2.674743621 | 0.981 | 0.373 | 1.84E-198 | 7 |
| Ly6c1 | 0 | 1.915405204 | 0.706 | 0.021 | 0 | 8 |
| Cldn5 | 0 | 1.685466667 | 0.545 | 0.009 | 0 | 8 |
| Calcrl | 0 | 1.553471693 | 0.657 | 0.033 | 0 | 8 |
| Ramp2 | 0 | 1.444878009 | 0.524 | 0.008 | 0 | 8 |
| Sparc | 0 | 1.434427245 | 0.636 | 0.019 | 0 | 8 |
| Ptprb | 0 | 1.346613901 | 0.469 | 0.006 | 0 | 8 |
| Cd36 | 0 | 1.301828093 | 0.587 | 0.011 | 0 | 8 |
| Epas1 | 0 | 1.295979202 | 0.545 | 0.022 | 0 | 8 |
| Ace | 0 | 1.271290893 | 0.503 | 0.01 | 0 | 8 |
| Ly6a | 8.77E-192 | 1.970385001 | 0.776 | 0.09 | 2.72E-187 | 8 |

**Table S2.** **Functional signatures with gene list.**

| Azurophil granules | Specific granules | Gelatinase granules | Secretory vesicles | NADPH oxidase | ROS production | Chemotaxis |
| --- | --- | --- | --- | --- | --- | --- |
| Hexa | Lyz2 | Ceacam1 | Cd63 | Cybb | Cyba | C5ar1 |
| Prss57 | Lcn2 | Mmp8 | Mme | Cyba | Cybb | Ccl1 |
| Prtn3 | Cyba | Mmp9 | Itgam | Rac2 | Cyp1a1 | Ccl11 |
| Ctsg | Cybb | Itgam | Fut4 | Rac1 | Cyp1a2 | Ccl12 |
| Elane | Ncf1 | Slc11a1 | Fcgr3 | Ncf2 | Cyp1b1 | Ccl17 |
| Mpo | Ncf4 |  | Cr2 | Ncf1 | Ddah1 | Ccl19 |
| Ctsc | Ltf |  | Cybb | Ncf4 | Duox1 | Ccl2 |
|  | Camp |  | Mmp25 |  | Duox2 | Ccl20 |
|  |  |  | Slc11a2 |  | Gbf1 | Ccl21a |
|  |  |  | Fpr1 |  | Hsp90aa1 | Ccl21b |
|  |  |  | Scamp1 |  | Mpo | Ccl21c |
|  |  |  | Vamp2 |  | Ncf1 | Ccl22 |
|  |  |  | Stxbp4 |  | Nos1 | Ccl24 |
|  |  |  | Cd93 |  | Nos2 | Ccl25 |
|  |  |  | Cr1l |  | Nos3 | Ccl26 |
|  |  |  |  |  | P2rx4 | Ccl3 |
|  |  |  |  |  | Rora | Ccl4 |
|  |  |  |  |  | Slc7a2 | Ccl5 |
|  |  |  |  |  | Sod1 | Ccl6 |
|  |  |  |  |  | Sod2 | Ccl7 |
|  |  |  |  |  | Spr | Ccl8 |
|  |  |  |  |  |  | Ccl9 |
|  |  |  |  |  |  | Cklf |
|  |  |  |  |  |  | Csf3r |
|  |  |  |  |  |  | Cx3cl1 |
|  |  |  |  |  |  | Cxadr |
|  |  |  |  |  |  | Cxcl1 |
|  |  |  |  |  |  | Cxcl10 |
|  |  |  |  |  |  | Cxcl13 |
|  |  |  |  |  |  | Cxcl15 |
|  |  |  |  |  |  | Cxcl2 |
|  |  |  |  |  |  | Cxcl3 |
|  |  |  |  |  |  | Cxcl5 |
|  |  |  |  |  |  | Cxcl9 |
|  |  |  |  |  |  | Cxcr1 |
|  |  |  |  |  |  | Cxcr2 |
|  |  |  |  |  |  | Edn3 |
|  |  |  |  |  |  | Fcer1g |
|  |  |  |  |  |  | Fcgr3 |
|  |  |  |  |  |  | Gbf1 |
|  |  |  |  |  |  | Gm2564 |
|  |  |  |  |  |  | Ifng |
|  |  |  |  |  |  | Il17b |
|  |  |  |  |  |  | Il1b |
|  |  |  |  |  |  | Il1f10 |
|  |  |  |  |  |  | Il1rn |
|  |  |  |  |  |  | Itga1 |
|  |  |  |  |  |  | Itga9 |
|  |  |  |  |  |  | Itgam |
|  |  |  |  |  |  | Itgb2 |
|  |  |  |  |  |  | Lgals3 |
|  |  |  |  |  |  | Nckap1l |
|  |  |  |  |  |  | Pde4b |
|  |  |  |  |  |  | Pde4d |
|  |  |  |  |  |  | Pf4 |
|  |  |  |  |  |  | Pla2g1b |
|  |  |  |  |  |  | Ppbp |
|  |  |  |  |  |  | Prex1 |
|  |  |  |  |  |  | Prkca |
|  |  |  |  |  |  | S100a8 |
|  |  |  |  |  |  | S100a9 |
|  |  |  |  |  |  | Slc37a4 |
|  |  |  |  |  |  | Spp1 |
|  |  |  |  |  |  | Syk |
|  |  |  |  |  |  | Tgfb2 |
|  |  |  |  |  |  | Trem1 |
|  |  |  |  |  |  | Trem3 |
|  |  |  |  |  |  | Vav1 |
|  |  |  |  |  |  | Vav3 |
|  |  |  |  |  |  | Xcl1 |

**Table S3.** **Results of differential gene expression analysis between LPS group and MSC group on day 3.**

| Gene | p_val | avg_logFC | pct.1 | pct.2 | p_val_adj | Foldchange |
| --- | --- | --- | --- | --- | --- | --- |
| Rpl37 | 0 | -0.367303855 | 0.872 | 0.923 | 0 | 0.692599163 |
| Rps29 | 0 | -0.450603997 | 0.946 | 0.978 | 0 | 0.637243142 |
| Rpl38 | 0 | -0.451085574 | 0.81 | 0.906 | 0 | 0.636936335 |
| Rps27 | 0 | -0.453432535 | 0.955 | 0.981 | 0 | 0.635443223 |
| Rpl35 | 0 | -0.490357132 | 0.539 | 0.725 | 0 | 0.612407645 |
| Rps21 | 9.31E-300 | -0.383246857 | 0.78 | 0.868 | 2.89E-295 | 0.68164461 |
| Rpl37a | 5.87E-298 | -0.353853782 | 0.869 | 0.919 | 1.82E-293 | 0.701977602 |
| Gbp2 | 1.16E-270 | 0.591508448 | 0.668 | 0.464 | 3.62E-266 | 1.806711691 |
| Isg15 | 4.01E-260 | 0.654247941 | 0.53 | 0.331 | 1.24E-255 | 1.92369524 |
| Gm10076 | 1.30E-253 | -0.319172687 | 0.055 | 0.211 | 4.03E-249 | 0.726750038 |
| Rpl36 | 1.66E-246 | -0.359250617 | 0.738 | 0.828 | 5.16E-242 | 0.698199349 |
| Psmb8 | 4.06E-224 | 0.47480409 | 0.693 | 0.538 | 1.26E-219 | 1.607699202 |
| Ifitm3 | 1.37E-223 | 0.533191305 | 0.757 | 0.627 | 4.26E-219 | 1.704362781 |
| AW112010 | 1.25E-222 | 0.885745372 | 0.465 | 0.273 | 3.89E-218 | 2.42479109 |
| Zbp1 | 1.73E-212 | 0.470426185 | 0.458 | 0.264 | 5.38E-208 | 1.600676232 |
| Samhd1 | 1.10E-198 | 0.421206403 | 0.675 | 0.519 | 3.40E-194 | 1.523798762 |
| Rps28 | 1.64E-195 | -0.327337627 | 0.761 | 0.843 | 5.10E-191 | 0.720840326 |
| Ifi47 | 2.06E-193 | 0.473914683 | 0.449 | 0.262 | 6.39E-189 | 1.606269939 |
| Rpl39 | 3.71E-189 | -0.322356969 | 0.718 | 0.803 | 1.15E-184 | 0.724439542 |
| Gbp5 | 6.79E-182 | 0.453628749 | 0.426 | 0.243 | 2.11E-177 | 1.574013535 |
| Oasl2 | 1.12E-174 | 0.412871802 | 0.419 | 0.245 | 3.47E-170 | 1.511151287 |
| Cxcl10 | 2.29E-164 | 0.576759315 | 0.434 | 0.257 | 7.11E-160 | 1.780259812 |
| Fcgr4 | 3.12E-161 | 0.39518013 | 0.556 | 0.393 | 9.68E-157 | 1.484651597 |
| Rtp4 | 9.14E-161 | 0.38436293 | 0.431 | 0.262 | 2.84E-156 | 1.468678372 |
| Rsad2 | 3.01E-159 | 0.516064524 | 0.334 | 0.178 | 9.33E-155 | 1.675421078 |
| Ly6i | 1.23E-150 | 0.50909268 | 0.558 | 0.424 | 3.83E-146 | 1.663780929 |
| Herc6 | 2.35E-144 | 0.335634249 | 0.278 | 0.137 | 7.30E-140 | 1.398827309 |
| Prdx5 | 2.40E-144 | 0.308018256 | 0.967 | 0.942 | 7.46E-140 | 1.36072583 |
| Trim30a | 2.64E-143 | 0.345663796 | 0.459 | 0.297 | 8.20E-139 | 1.412927504 |
| Parp14 | 3.82E-142 | 0.354032314 | 0.556 | 0.397 | 1.19E-137 | 1.424801227 |
| Gbp7 | 2.09E-139 | 0.355624163 | 0.515 | 0.349 | 6.50E-135 | 1.427071101 |
| Stat1 | 4.70E-137 | 0.34006126 | 0.624 | 0.47 | 1.46E-132 | 1.405033661 |
| Irgm1 | 1.60E-134 | 0.309307736 | 0.363 | 0.21 | 4.97E-130 | 1.36248159 |
| Rps26 | 1.44E-133 | -0.236686542 | 0.849 | 0.9 | 4.46E-129 | 0.789238642 |
| Hspa8 | 1.68E-129 | 0.278600999 | 0.894 | 0.841 | 5.21E-125 | 1.321280047 |
| Irf1 | 4.50E-129 | 0.319101909 | 0.644 | 0.493 | 1.40E-124 | 1.375891533 |
| Mrpl52 | 2.50E-128 | -0.283300273 | 0.711 | 0.779 | 7.76E-124 | 0.75329356 |
| Psmb9 | 3.35E-128 | 0.314250094 | 0.546 | 0.392 | 1.04E-123 | 1.369232131 |
| Psme2 | 8.21E-127 | 0.318535796 | 0.703 | 0.593 | 2.55E-122 | 1.375112844 |
| Tap2 | 6.75E-124 | 0.312590417 | 0.493 | 0.347 | 2.10E-119 | 1.366961532 |
| Rpl34 | 1.02E-123 | -0.206077209 | 0.918 | 0.94 | 3.17E-119 | 0.813770243 |
| Ifi204 | 6.11E-122 | 0.371549722 | 0.413 | 0.271 | 1.90E-117 | 1.449979941 |
| Uba52 | 6.38E-122 | -0.28930527 | 0.255 | 0.383 | 1.98E-117 | 0.748783589 |
| Rpl10 | 8.03E-122 | -0.227539612 | 0.821 | 0.866 | 2.49E-117 | 0.79649087 |
| Il18bp | 1.92E-121 | 0.31599709 | 0.346 | 0.204 | 5.96E-117 | 1.371626265 |
| Irf7 | 3.68E-120 | 0.311967843 | 0.286 | 0.156 | 1.14E-115 | 1.366110762 |
| Igtp | 1.24E-119 | 0.308419494 | 0.423 | 0.271 | 3.86E-115 | 1.361271915 |
| H2-D1 | 8.37E-117 | 0.215359768 | 0.961 | 0.938 | 2.60E-112 | 1.24030804 |
| Rpl35a | 3.47E-115 | -0.191878173 | 0.95 | 0.963 | 1.08E-110 | 0.825407419 |
| Pla2g7 | 3.74E-115 | 0.362901153 | 0.6 | 0.475 | 1.16E-110 | 1.43749376 |
| Gbp3 | 4.27E-113 | 0.311021166 | 0.267 | 0.142 | 1.33E-108 | 1.364818109 |
| Tap1 | 7.29E-113 | 0.287500936 | 0.515 | 0.367 | 2.26E-108 | 1.333091841 |
| Ifit1 | 2.65E-112 | 0.406089971 | 0.166 | 0.068 | 8.22E-108 | 1.500937588 |
| Nos2 | 5.17E-111 | 0.421915874 | 0.287 | 0.164 | 1.61E-106 | 1.524880238 |
| Eno1 | 1.38E-106 | -0.243785231 | 0.505 | 0.587 | 4.28E-102 | 0.783655922 |
| Psmb10 | 2.20E-106 | 0.300767093 | 0.488 | 0.352 | 6.83E-102 | 1.350894673 |
| Xaf1 | 3.64E-106 | 0.269092058 | 0.233 | 0.12 | 1.13E-101 | 1.308775618 |
| Eif1 | 2.24E-104 | -0.192955023 | 0.995 | 0.997 | 6.97E-100 | 0.824519058 |
| Lyz2 | 2.37E-101 | -0.289565827 | 0.939 | 0.957 | 7.35E-97 | 0.748588514 |
| Ddit3 | 1.97E-100 | -0.416215091 | 0.349 | 0.439 | 6.12E-96 | 0.659538395 |
| H2-T23 | 3.47E-98 | 0.281139622 | 0.523 | 0.397 | 1.08E-93 | 1.32463854 |
| Rps15 | 1.37E-95 | -0.213565502 | 0.774 | 0.834 | 4.26E-91 | 0.807699253 |
| Rps12 | 8.46E-95 | -0.193583186 | 0.934 | 0.954 | 2.63E-90 | 0.824001288 |
| Ctss | 2.86E-94 | 0.29261087 | 0.626 | 0.513 | 8.87E-90 | 1.339921285 |
| Tapbp | 3.11E-94 | 0.252308735 | 0.715 | 0.623 | 9.65E-90 | 1.286993315 |
| Slfn1 | 6.70E-94 | 0.266292359 | 0.679 | 0.556 | 2.08E-89 | 1.305116566 |
| Hp | 5.31E-93 | 0.300308675 | 0.84 | 0.76 | 1.65E-88 | 1.35027554 |
| Slfn4 | 2.93E-92 | 0.362897135 | 0.381 | 0.261 | 9.09E-88 | 1.437487984 |
| Rpl30 | 1.31E-90 | -0.193612786 | 0.885 | 0.911 | 4.08E-86 | 0.823976898 |
| Tnf | 3.38E-88 | 0.390925955 | 0.626 | 0.532 | 1.05E-83 | 1.478349044 |
| Bst2 | 1.18E-86 | 0.296544876 | 0.419 | 0.302 | 3.65E-82 | 1.345202926 |
| Lars2 | 1.49E-86 | 0.223421991 | 0.911 | 0.863 | 4.63E-82 | 1.250348098 |
| Rps15a | 2.78E-86 | -0.194858494 | 0.837 | 0.871 | 8.64E-82 | 0.822951102 |
| Plac8 | 2.30E-85 | 0.398875396 | 0.46 | 0.337 | 7.14E-81 | 1.490147929 |
| Trafd1 | 4.01E-84 | 0.241334157 | 0.271 | 0.163 | 1.25E-79 | 1.272946328 |
| Tgtp1 | 1.56E-83 | 0.226539613 | 0.127 | 0.051 | 4.85E-79 | 1.254252294 |
| Atp5e | 4.85E-83 | -0.193811249 | 0.706 | 0.763 | 1.50E-78 | 0.823813385 |
| Icam1 | 2.82E-81 | 0.251278547 | 0.697 | 0.597 | 8.75E-77 | 1.285668153 |
| Oas3 | 3.20E-81 | 0.209607179 | 0.217 | 0.119 | 9.95E-77 | 1.23319354 |
| Gstm1 | 2.70E-80 | -0.379333781 | 0.203 | 0.308 | 8.38E-76 | 0.684317162 |
| H2-K1 | 5.82E-79 | 0.259501536 | 0.871 | 0.833 | 1.81E-74 | 1.296283775 |
| Rps17 | 1.24E-78 | -0.196502155 | 0.698 | 0.756 | 3.84E-74 | 0.82159956 |
| Psme1 | 1.02E-77 | 0.257754053 | 0.551 | 0.436 | 3.17E-73 | 1.294020519 |
| Isg20 | 2.14E-74 | 0.26621506 | 0.295 | 0.191 | 6.65E-70 | 1.305015685 |
| Pnp | 6.59E-74 | 0.231738048 | 0.677 | 0.586 | 2.05E-69 | 1.26078942 |
| Lcn2 | 2.30E-73 | 0.189344075 | 0.987 | 0.977 | 7.14E-69 | 1.208456681 |
| Bola2 | 3.01E-71 | -0.231151877 | 0.287 | 0.391 | 9.36E-67 | 0.793618924 |
| Rpl22 | 5.55E-71 | -0.191273771 | 0.693 | 0.749 | 1.72E-66 | 0.825906448 |
| Ehd1 | 2.39E-69 | 0.194301511 | 0.809 | 0.745 | 7.41E-65 | 1.214462401 |
| Ube2l6 | 2.89E-69 | 0.214238028 | 0.224 | 0.133 | 8.97E-65 | 1.238917517 |
| Sp100 | 4.43E-69 | 0.217633373 | 0.331 | 0.224 | 1.38E-64 | 1.243131219 |
| mt-Nd3 | 5.59E-69 | -0.248202472 | 0.376 | 0.473 | 1.74E-64 | 0.780201958 |
| Eea1 | 7.86E-69 | -0.302207157 | 0.287 | 0.371 | 2.44E-64 | 0.739184922 |
| Cflar | 1.78E-68 | 0.205275389 | 0.731 | 0.651 | 5.54E-64 | 1.227863158 |
| Chil3 | 1.24E-67 | 0.363111045 | 0.857 | 0.805 | 3.87E-63 | 1.437795511 |
| Ifi211 | 6.77E-67 | 0.202786676 | 0.128 | 0.059 | 2.10E-62 | 1.224811158 |
| Acod1 | 1.29E-66 | 0.21996486 | 0.812 | 0.748 | 4.02E-62 | 1.246032944 |
| Cd274 | 2.89E-66 | 0.22150889 | 0.724 | 0.635 | 8.97E-62 | 1.247958343 |
| Slfn5 | 3.22E-66 | 0.283922355 | 0.196 | 0.113 | 9.99E-62 | 1.328329789 |
| Spp1 | 1.18E-65 | -0.673796287 | 0.155 | 0.241 | 3.65E-61 | 0.509769668 |
| Ftl1-ps1 | 2.98E-65 | 0.234579656 | 0.656 | 0.58 | 9.25E-61 | 1.264377184 |
| B2m | 3.89E-65 | 0.188411025 | 0.973 | 0.969 | 1.21E-60 | 1.207329656 |
| Trim12c | 9.45E-64 | 0.192365384 | 0.312 | 0.209 | 2.93E-59 | 1.212113323 |
| Atf4 | 1.13E-63 | -0.236436221 | 0.677 | 0.723 | 3.52E-59 | 0.78943623 |
| Rpl12 | 1.87E-63 | -0.212906719 | 0.625 | 0.687 | 5.79E-59 | 0.808231526 |
| Hgsnat | 3.54E-62 | -0.395260295 | 0.227 | 0.307 | 1.10E-57 | 0.673504706 |
| Ly6e | 5.26E-62 | 0.223700574 | 0.622 | 0.523 | 1.63E-57 | 1.250696472 |
| Tifa | 6.47E-62 | 0.227861871 | 0.298 | 0.2 | 2.01E-57 | 1.255911835 |
| Nampt | 1.35E-61 | 0.246474682 | 0.477 | 0.376 | 4.18E-57 | 1.279506788 |
| H2-Q4 | 2.38E-61 | 0.225378982 | 0.389 | 0.285 | 7.39E-57 | 1.252797414 |
| Tspo | 1.36E-59 | 0.224668153 | 0.755 | 0.69 | 4.21E-55 | 1.251907206 |
| Trim30b | 1.60E-59 | 0.206844376 | 0.374 | 0.27 | 4.98E-55 | 1.229791172 |
| Pet100 | 2.57E-59 | -0.198065975 | 0.346 | 0.426 | 7.97E-55 | 0.820315731 |
| Ccl5 | 5.86E-59 | 0.194903833 | 0.179 | 0.101 | 1.82E-54 | 1.215194119 |
| Snx10 | 1.22E-58 | 0.187036346 | 0.685 | 0.597 | 3.79E-54 | 1.205671105 |
| Rnaset2a | 3.64E-58 | 0.209112099 | 0.366 | 0.269 | 1.13E-53 | 1.232583162 |
| Romo1 | 9.07E-57 | -0.197336272 | 0.31 | 0.397 | 2.82E-52 | 0.820914537 |
| Clec4d | 5.04E-56 | -0.190184902 | 0.92 | 0.936 | 1.57E-51 | 0.826806242 |
| Lst1 | 1.78E-55 | -0.20744342 | 0.794 | 0.808 | 5.53E-51 | 0.812659221 |
| Cstb | 2.43E-55 | -0.251721001 | 0.909 | 0.933 | 7.55E-51 | 0.777461619 |
| Itpr2 | 5.38E-55 | -0.454773828 | 0.378 | 0.439 | 1.67E-50 | 0.634591478 |
| Atf5 | 1.39E-54 | -0.306434022 | 0.204 | 0.281 | 4.31E-50 | 0.736067081 |
| Nme1 | 9.44E-54 | -0.221350268 | 0.323 | 0.401 | 2.93E-49 | 0.801435914 |
| Atp5k | 4.02E-53 | -0.184087327 | 0.347 | 0.422 | 1.25E-48 | 0.831863156 |
| Socs3 | 6.68E-53 | 0.184732449 | 0.782 | 0.713 | 2.08E-48 | 1.20289656 |
| Dnaja1 | 7.06E-53 | 0.213773382 | 0.686 | 0.612 | 2.19E-48 | 1.238341992 |
| Mrgpra2b | 1.10E-52 | 0.216818525 | 0.338 | 0.243 | 3.40E-48 | 1.242118669 |
| Sars | 3.29E-52 | -0.210991909 | 0.194 | 0.266 | 1.02E-47 | 0.809780619 |
| Marcksl1 | 8.57E-52 | 0.209833741 | 0.735 | 0.668 | 2.66E-47 | 1.233472967 |
| Odc1 | 6.23E-51 | -0.294753453 | 0.205 | 0.283 | 1.94E-46 | 0.744715172 |
| Fam26f | 3.00E-50 | 0.213299575 | 0.129 | 0.067 | 9.31E-46 | 1.237755397 |
| Gm14005 | 3.17E-50 | -0.216584859 | 0.29 | 0.356 | 9.86E-46 | 0.805264198 |
| Sell | 7.98E-50 | 0.191331528 | 0.381 | 0.284 | 2.48E-45 | 1.21086082 |
| Sppl2a | 4.89E-49 | 0.194128333 | 0.448 | 0.357 | 1.52E-44 | 1.214252101 |
| Wfdc17 | 7.00E-48 | 0.204257568 | 0.903 | 0.864 | 2.17E-43 | 1.22661405 |
| Eif2s2 | 6.49E-47 | -0.185141423 | 0.607 | 0.658 | 2.01E-42 | 0.830986755 |
| Prdx1 | 2.16E-45 | -0.230345573 | 0.421 | 0.5 | 6.71E-41 | 0.794259081 |
| Atf3 | 8.04E-45 | -0.262292503 | 0.398 | 0.465 | 2.50E-40 | 0.769285972 |
| AA467197 | 1.69E-44 | 0.240718246 | 0.834 | 0.798 | 5.25E-40 | 1.272162548 |
| Batf | 3.58E-44 | 0.182326626 | 0.49 | 0.399 | 1.11E-39 | 1.200006083 |
| Alcam | 7.94E-44 | -0.20107412 | 0.237 | 0.31 | 2.46E-39 | 0.81785181 |
| Lgals3 | 1.44E-43 | -0.235702523 | 0.774 | 0.811 | 4.46E-39 | 0.79001565 |
| Mdm2 | 1.57E-43 | -0.421245806 | 0.492 | 0.535 | 4.88E-39 | 0.656228777 |
| Smpdl3b | 1.05E-42 | 0.185881116 | 0.277 | 0.201 | 3.28E-38 | 1.204279082 |
| Glipr1 | 1.60E-42 | -0.194114542 | 0.221 | 0.291 | 4.97E-38 | 0.823563566 |
| Il1a | 9.23E-42 | 0.213887603 | 0.753 | 0.694 | 2.87E-37 | 1.238483445 |
| Il1r2 | 3.66E-41 | 0.217944004 | 0.544 | 0.461 | 1.14E-36 | 1.243517433 |
| Gm15340 | 1.60E-39 | -0.205043209 | 0.117 | 0.173 | 4.98E-35 | 0.814612117 |
| Creb5 | 1.90E-39 | -0.210825118 | 0.299 | 0.37 | 5.90E-35 | 0.809915694 |
| Ifitm6 | 3.72E-39 | 0.353123406 | 0.212 | 0.15 | 1.15E-34 | 1.423506802 |
| Eif3c | 1.37E-38 | -0.188200002 | 0.447 | 0.496 | 4.27E-34 | 0.828448999 |
| Retnlg | 1.67E-37 | 0.439945216 | 0.387 | 0.307 | 5.19E-33 | 1.552622158 |
| 1700017B05Rik | 3.56E-36 | -0.208754935 | 0.287 | 0.348 | 1.11E-31 | 0.811594105 |
| E030030I06Rik | 4.16E-36 | -0.196274428 | 0.295 | 0.345 | 1.29E-31 | 0.821786682 |
| Hspa9 | 4.28E-36 | -0.187162071 | 0.247 | 0.312 | 1.33E-31 | 0.829309319 |
| Txnip | 4.70E-36 | 0.189264174 | 0.484 | 0.401 | 1.46E-31 | 1.208360128 |
| Asprv1 | 8.39E-36 | 0.298531433 | 0.539 | 0.471 | 2.61E-31 | 1.347877904 |
| Krtcap2 | 2.02E-35 | -0.206804305 | 0.221 | 0.285 | 6.28E-31 | 0.813178769 |
| Mgst1 | 1.79E-33 | 0.185796861 | 0.29 | 0.22 | 5.57E-29 | 1.204177621 |
| Dedd2 | 5.09E-33 | -0.225311821 | 0.291 | 0.336 | 1.58E-28 | 0.798267263 |
| Cyp4f18 | 8.99E-33 | -0.318209037 | 0.399 | 0.43 | 2.79E-28 | 0.727450708 |
| E230032D23Rik | 1.39E-32 | -0.186544982 | 0.294 | 0.352 | 4.30E-28 | 0.829821234 |
| Dstn | 5.88E-32 | 0.190608569 | 0.554 | 0.485 | 1.83E-27 | 1.209985734 |
| Cybb | 4.40E-31 | 0.2084596 | 0.523 | 0.452 | 1.37E-26 | 1.231779165 |
| Ifitm1 | 4.49E-31 | 0.207194752 | 0.68 | 0.607 | 1.40E-26 | 1.230222136 |
| Gm12840 | 9.24E-28 | 0.206006204 | 0.311 | 0.244 | 2.87E-23 | 1.228760827 |
| Hist1h1c | 1.58E-26 | -0.187562584 | 0.177 | 0.233 | 4.90E-22 | 0.828977236 |
| Gadd45g | 7.21E-21 | -0.330889655 | 0.142 | 0.17 | 2.24E-16 | 0.718284424 |
| G0s2 | 2.14E-16 | 0.208660444 | 0.522 | 0.475 | 6.64E-12 | 1.232026586 |
| Stfa2 | 4.08E-15 | 0.217930219 | 0.183 | 0.142 | 1.27E-10 | 1.243500292 |
| BC100530 | 4.19E-14 | 0.337306743 | 0.167 | 0.13 | 1.30E-09 | 1.401168797 |
| Rgs1 | 1.15E-12 | -0.208035626 | 0.294 | 0.329 | 3.56E-08 | 0.812178102 |
| Stfa1 | 6.34E-08 | 0.184352754 | 0.132 | 0.106 | 0.001969071 | 1.202439914 |

**Table S4. Results of differential gene expression analysis between LPS group and MSC group on day 7.**

| Gene | p_val | avg_logFC | pct.1 | pct.2 | p_val_adj | Foldchange |
| --- | --- | --- | --- | --- | --- | --- |
| Rbm3 | 0 | 0.932660969 | 0.912 | 0.569 | 0 | 2.54126241 |
| Malat1 | 0 | -0.457911533 | 0.998 | 0.997 | 0 | 0.632603438 |
| Lyz2 | 0 | -0.630704419 | 0.99 | 0.999 | 0 | 0.532216765 |
| Uba52 | 0 | -0.664770271 | 0.594 | 0.785 | 0 | 0.514391685 |
| Cd74 | 0 | -0.785937998 | 0.328 | 0.647 | 0 | 0.455692063 |
| Chil3 | 4.94E-324 | 0.831314145 | 0.896 | 0.666 | 1.53E-319 | 2.296334475 |
| Cirbp | 4.83E-262 | 0.589900469 | 0.496 | 0.191 | 1.50E-257 | 1.803808871 |
| Apoe | 1.11E-201 | -0.288665647 | 0.923 | 0.98 | 3.44E-197 | 0.749262682 |
| H2-Ab1 | 2.33E-167 | -0.541206341 | 0.121 | 0.312 | 7.24E-163 | 0.582045683 |
| Irf1 | 3.86E-165 | 0.544654097 | 0.653 | 0.411 | 1.20E-160 | 1.724011937 |
| Lst1 | 6.46E-165 | -0.467319692 | 0.756 | 0.844 | 2.00E-160 | 0.626679714 |
| H2-Eb1 | 4.53E-163 | -0.509042549 | 0.095 | 0.284 | 1.41E-158 | 0.601070799 |
| Hist1h2bc | 9.09E-155 | -0.602965144 | 0.252 | 0.437 | 2.82E-150 | 0.547186741 |
| Saa3 | 7.06E-153 | 0.22428933 | 0.991 | 0.933 | 2.19E-148 | 1.251433044 |
| Hexa | 6.59E-152 | -0.496937879 | 0.335 | 0.476 | 2.05E-147 | 0.608390777 |
| Id2 | 5.48E-149 | 0.594598471 | 0.618 | 0.389 | 1.70E-144 | 1.812303107 |
| H2-Aa | 1.99E-144 | -0.506359576 | 0.115 | 0.288 | 6.18E-140 | 0.602685621 |
| Ssbp4 | 3.34E-135 | 0.401954021 | 0.362 | 0.157 | 1.04E-130 | 1.494742605 |
| Hcst | 3.42E-133 | 0.393442665 | 0.67 | 0.448 | 1.06E-128 | 1.482074306 |
| Hist1h1c | 8.29E-123 | -0.603795752 | 0.194 | 0.36 | 2.57E-118 | 0.546732432 |
| Ptprc | 1.04E-121 | -0.376738678 | 0.771 | 0.808 | 3.21E-117 | 0.686095342 |
| Ctsd | 1.05E-121 | -0.352220712 | 0.894 | 0.94 | 3.25E-117 | 0.703124916 |
| Cfl1 | 1.30E-120 | 0.270692673 | 0.944 | 0.872 | 4.05E-116 | 1.310872142 |
| Rsrp1 | 3.85E-120 | 0.349011418 | 0.83 | 0.686 | 1.20E-115 | 1.417665377 |
| Hsp90ab1 | 1.53E-119 | -0.413171038 | 0.591 | 0.688 | 4.75E-115 | 0.661549123 |
| Tap1 | 1.63E-119 | 0.428461019 | 0.436 | 0.23 | 5.05E-115 | 1.534893533 |
| Irgm1 | 1.78E-117 | 0.431667628 | 0.298 | 0.121 | 5.53E-113 | 1.539823236 |
| Eif1 | 1.40E-111 | -0.255700546 | 0.986 | 0.987 | 4.35E-107 | 0.774373823 |
| Igtp | 2.93E-109 | 0.458379151 | 0.319 | 0.143 | 9.09E-105 | 1.58150852 |
| Hist1h4i | 3.95E-109 | -0.516032222 | 0.119 | 0.255 | 1.23E-104 | 0.596884159 |
| Ccl6 | 2.75E-106 | -0.429199969 | 0.456 | 0.657 | 8.53E-102 | 0.651029731 |
| Gbp2 | 4.30E-106 | 0.423330652 | 0.497 | 0.294 | 1.34E-101 | 1.527039131 |
| mt-Co2 | 5.51E-106 | 0.242400144 | 0.976 | 0.905 | 1.71E-101 | 1.274303996 |
| Nlrp3 | 1.97E-105 | -0.483016225 | 0.591 | 0.704 | 6.12E-101 | 0.616919814 |
| Atf3 | 7.02E-105 | -0.589394482 | 0.297 | 0.432 | 2.18E-100 | 0.554663042 |
| Psmb8 | 2.21E-104 | 0.447815472 | 0.602 | 0.421 | 6.86E-100 | 1.564889903 |
| Fcgr4 | 4.26E-104 | 0.478110818 | 0.476 | 0.287 | 1.32E-99 | 1.613024225 |
| Hexb | 5.78E-102 | 0.425291093 | 0.593 | 0.404 | 1.79E-97 | 1.530035737 |
| Clec4d | 1.54E-96 | -0.305341636 | 0.857 | 0.9 | 4.79E-92 | 0.736871589 |
| Rpl41 | 1.05E-95 | 0.197448151 | 0.992 | 0.975 | 3.26E-91 | 1.218289896 |
| Gm10076 | 1.12E-93 | -0.346379244 | 0.37 | 0.477 | 3.47E-89 | 0.707244218 |
| Tax1bp1 | 1.75E-93 | -0.293332958 | 0.652 | 0.706 | 5.44E-89 | 0.745773788 |
| Prdx5 | 7.78E-93 | 0.368255753 | 0.953 | 0.897 | 2.42E-88 | 1.445211608 |
| Zbp1 | 6.23E-92 | 0.430105815 | 0.357 | 0.188 | 1.93E-87 | 1.537420196 |
| Cox5b | 8.47E-91 | 0.283541374 | 0.815 | 0.685 | 2.63E-86 | 1.327823817 |
| Tmsb10 | 7.02E-90 | -0.43639766 | 0.429 | 0.571 | 2.18E-85 | 0.646360643 |
| Cyba | 1.09E-88 | 0.277514709 | 0.976 | 0.955 | 3.39E-84 | 1.319845533 |
| Ifi47 | 8.23E-88 | 0.420170623 | 0.289 | 0.134 | 2.56E-83 | 1.522221259 |
| Slc7a11 | 2.56E-84 | -0.423482191 | 0.534 | 0.656 | 7.96E-80 | 0.654762836 |
| Csf3r | 3.30E-84 | -0.356366619 | 0.663 | 0.749 | 1.03E-79 | 0.700215861 |
| Klhl6 | 4.86E-83 | 0.369395493 | 0.368 | 0.202 | 1.51E-78 | 1.446859713 |
| Tmsb4x | 1.72E-80 | -0.269750877 | 0.999 | 1 | 5.35E-76 | 0.763569693 |
| Tapbp | 1.75E-79 | 0.324362967 | 0.696 | 0.547 | 5.44E-75 | 1.383149253 |
| Grina | 2.58E-78 | 0.246284307 | 0.919 | 0.837 | 8.00E-74 | 1.279263226 |
| Ptgs2 | 2.01E-77 | -0.434623387 | 0.524 | 0.659 | 6.24E-73 | 0.647508482 |
| Anxa2 | 1.06E-76 | -0.317221193 | 0.632 | 0.687 | 3.31E-72 | 0.728169671 |
| Serinc3 | 1.64E-76 | 0.287068629 | 0.758 | 0.616 | 5.08E-72 | 1.33251566 |
| H2-K1 | 2.69E-76 | 0.347056332 | 0.916 | 0.863 | 8.34E-72 | 1.414896427 |
| Sh3bgrl3 | 4.59E-76 | 0.220609831 | 0.969 | 0.925 | 1.43E-71 | 1.246836859 |
| Crlf2 | 1.24E-75 | 0.312059797 | 0.533 | 0.364 | 3.84E-71 | 1.366236387 |
| Serf2 | 1.44E-75 | -0.211551973 | 0.911 | 0.919 | 4.48E-71 | 0.809327217 |
| Tmem128 | 3.47E-75 | 0.274686725 | 0.332 | 0.179 | 1.08E-70 | 1.316118303 |
| Il1f9 | 4.67E-75 | -0.44414332 | 0.225 | 0.372 | 1.45E-70 | 0.641373493 |
| Gm34084 | 5.04E-75 | -0.336900451 | 0.076 | 0.166 | 1.57E-70 | 0.713979913 |
| Hnrnpc | 7.50E-75 | -0.306823517 | 0.386 | 0.486 | 2.33E-70 | 0.735780442 |
| Gna13 | 4.05E-74 | 0.280870221 | 0.486 | 0.316 | 1.26E-69 | 1.324281728 |
| Pkm | 2.84E-73 | 0.247580113 | 0.84 | 0.727 | 8.83E-69 | 1.280921976 |
| Cd274 | 5.37E-73 | 0.30881796 | 0.593 | 0.423 | 1.67E-68 | 1.361814444 |
| Lcn2 | 1.59E-72 | 0.211480885 | 0.946 | 0.857 | 4.93E-68 | 1.235506348 |
| Srsf5 | 1.99E-71 | -0.230111355 | 0.743 | 0.772 | 6.17E-67 | 0.794445132 |
| Rps27rt | 1.12E-70 | 0.254235617 | 0.557 | 0.387 | 3.49E-66 | 1.289475591 |
| Prnp | 1.55E-70 | 0.370364548 | 0.328 | 0.183 | 4.82E-66 | 1.448262479 |
| AA467197 | 1.61E-70 | 0.421449275 | 0.753 | 0.63 | 5.01E-66 | 1.524168895 |
| Psmb3 | 1.25E-69 | 0.293397271 | 0.616 | 0.463 | 3.89E-65 | 1.340975415 |
| Tnf | 1.66E-69 | 0.429407743 | 0.445 | 0.286 | 5.16E-65 | 1.536347342 |
| Fpr2 | 2.08E-69 | 0.341939607 | 0.663 | 0.514 | 6.45E-65 | 1.407675281 |
| Ell2 | 8.11E-69 | -0.367544466 | 0.228 | 0.34 | 2.52E-64 | 0.692432536 |
| Psma2 | 1.05E-68 | 0.263973586 | 0.601 | 0.437 | 3.27E-64 | 1.302093803 |
| Lilrb4a | 2.81E-68 | -0.341220306 | 0.264 | 0.37 | 8.72E-64 | 0.710902275 |
| Plp2 | 3.60E-67 | 0.279860862 | 0.501 | 0.338 | 1.12E-62 | 1.322945727 |
| Psmb2 | 4.51E-67 | 0.263047557 | 0.483 | 0.321 | 1.40E-62 | 1.300888584 |
| 1110008P14Rik | 1.15E-66 | 0.273749344 | 0.53 | 0.367 | 3.58E-62 | 1.314885177 |
| Pip5k1c | 1.94E-66 | 0.252919405 | 0.341 | 0.196 | 6.02E-62 | 1.287779484 |
| Gm5617 | 6.02E-66 | 0.276650719 | 0.306 | 0.166 | 1.87E-61 | 1.318705692 |
| Ppia | 2.51E-65 | 0.227153111 | 0.907 | 0.811 | 7.80E-61 | 1.255022011 |
| Cxcl2 | 1.65E-64 | -0.293874784 | 0.952 | 0.982 | 5.11E-60 | 0.745369817 |
| Gapdh | 4.14E-64 | 0.212674138 | 0.942 | 0.882 | 1.29E-59 | 1.236981501 |
| Ly6i | 5.04E-64 | 0.38038132 | 0.48 | 0.325 | 1.57E-59 | 1.462842294 |
| Cd302 | 6.57E-64 | 0.294078852 | 0.428 | 0.275 | 2.04E-59 | 1.34188971 |
| mt-Atp6 | 7.82E-64 | 0.183645947 | 0.98 | 0.929 | 2.43E-59 | 1.201590322 |
| Wfdc17 | 2.88E-63 | 0.335260304 | 0.921 | 0.839 | 8.95E-59 | 1.398304322 |
| Ncf4 | 3.72E-63 | 0.254769461 | 0.722 | 0.581 | 1.15E-58 | 1.290164153 |
| AW112010 | 5.09E-63 | 0.431007238 | 0.506 | 0.351 | 1.58E-58 | 1.538806687 |
| Atp5g1 | 6.23E-63 | 0.289533652 | 0.53 | 0.378 | 1.93E-58 | 1.335804393 |
| Gbp7 | 2.13E-62 | 0.344370617 | 0.381 | 0.236 | 6.60E-58 | 1.411101517 |
| F10 | 3.34E-62 | 0.387899159 | 0.507 | 0.366 | 1.04E-57 | 1.473881149 |
| Plek | 5.65E-62 | -0.289851968 | 0.741 | 0.771 | 1.76E-57 | 0.748374343 |
| Trib1 | 6.99E-62 | -0.292460624 | 0.508 | 0.59 | 2.17E-57 | 0.746424636 |
| Irf7 | 1.02E-61 | 0.276419443 | 0.263 | 0.136 | 3.17E-57 | 1.318400742 |
| Socs3 | 9.64E-61 | 0.283770653 | 0.724 | 0.587 | 2.99E-56 | 1.328128294 |
| Zfp787 | 1.02E-60 | 0.24577715 | 0.267 | 0.141 | 3.16E-56 | 1.278614603 |
| Tcirg1 | 1.07E-60 | 0.27176144 | 0.408 | 0.26 | 3.33E-56 | 1.312273908 |
| Ddit3 | 1.34E-60 | -0.406283557 | 0.259 | 0.317 | 4.15E-56 | 0.666121257 |
| Spp1 | 3.53E-60 | -0.706243803 | 0.192 | 0.301 | 1.10E-55 | 0.493494382 |
| Dazap2 | 4.47E-60 | 0.207919063 | 0.812 | 0.688 | 1.39E-55 | 1.231113523 |
| Atf5 | 5.77E-60 | -0.367275493 | 0.046 | 0.112 | 1.79E-55 | 0.692618807 |
| Insig1 | 7.08E-60 | 0.27776502 | 0.189 | 0.082 | 2.20E-55 | 1.320175946 |
| Ctsc | 7.16E-60 | -0.353722753 | 0.218 | 0.304 | 2.22E-55 | 0.702069587 |
| Pla2g7 | 2.30E-59 | 0.234155882 | 0.633 | 0.478 | 7.13E-55 | 1.263841487 |
| Srp9 | 4.76E-59 | 0.241140867 | 0.603 | 0.451 | 1.48E-54 | 1.272700304 |
| Ndufs8 | 5.07E-59 | 0.24806896 | 0.273 | 0.146 | 1.58E-54 | 1.281548305 |
| Snrpb | 1.34E-58 | 0.206958023 | 0.586 | 0.431 | 4.15E-54 | 1.229930942 |
| Sorl1 | 1.53E-58 | -0.327912957 | 0.384 | 0.474 | 4.74E-54 | 0.720425725 |
| Drap1 | 2.86E-58 | 0.238067506 | 0.466 | 0.316 | 8.89E-54 | 1.268794841 |
| Cd33 | 3.27E-58 | 0.265014477 | 0.727 | 0.594 | 1.02E-53 | 1.303449846 |
| Sdhaf1 | 7.91E-58 | 0.268315667 | 0.261 | 0.138 | 2.46E-53 | 1.307759891 |
| Sell | 1.13E-57 | -0.356483888 | 0.257 | 0.353 | 3.49E-53 | 0.700133752 |
| AY036118 | 1.15E-57 | 0.235328384 | 0.652 | 0.501 | 3.58E-53 | 1.265324212 |
| Efhd2 | 1.34E-57 | 0.210471424 | 0.772 | 0.639 | 4.17E-53 | 1.234259782 |
| Rpl27 | 1.57E-57 | 0.205993112 | 0.858 | 0.756 | 4.88E-53 | 1.22874474 |
| Samhd1 | 2.62E-57 | 0.302529432 | 0.69 | 0.559 | 8.15E-53 | 1.353277505 |
| H2-T22 | 9.03E-57 | 0.264736865 | 0.25 | 0.13 | 2.80E-52 | 1.303088042 |
| Fam174a | 1.38E-56 | 0.248575858 | 0.432 | 0.287 | 4.28E-52 | 1.282198084 |
| Arf5 | 1.53E-56 | 0.207553331 | 0.745 | 0.611 | 4.76E-52 | 1.230663347 |
| Capns1 | 1.63E-56 | 0.207687601 | 0.497 | 0.348 | 5.06E-52 | 1.230828599 |
| Eif5a | 3.59E-56 | 0.236127829 | 0.737 | 0.608 | 1.12E-51 | 1.266336174 |
| Cd24a | 3.72E-56 | 0.259304711 | 0.786 | 0.666 | 1.16E-51 | 1.296028659 |
| Lamp1 | 6.27E-56 | -0.261112679 | 0.717 | 0.72 | 1.95E-51 | 0.77019413 |
| Rpl38 | 1.10E-55 | 0.186256302 | 0.958 | 0.914 | 3.41E-51 | 1.204730995 |
| Stap1 | 1.22E-55 | -0.35037784 | 0.112 | 0.206 | 3.80E-51 | 0.704421881 |
| Crip1 | 3.99E-55 | -0.314082094 | 0.297 | 0.415 | 1.24E-50 | 0.73045906 |
| Rnaset2a | 4.43E-55 | 0.20840231 | 0.272 | 0.151 | 1.37E-50 | 1.231708598 |
| H2-T23 | 5.45E-55 | 0.298893794 | 0.582 | 0.445 | 1.69E-50 | 1.348366411 |
| Stat1 | 5.96E-55 | 0.229499333 | 0.497 | 0.349 | 1.85E-50 | 1.257970029 |
| Lcp1 | 7.29E-55 | -0.205065235 | 0.858 | 0.871 | 2.26E-50 | 0.814594175 |
| Vdac2 | 8.98E-55 | 0.229898708 | 0.575 | 0.428 | 2.79E-50 | 1.258472531 |
| Dgat1 | 9.83E-55 | -0.352213956 | 0.242 | 0.34 | 3.05E-50 | 0.703129667 |
| Cflar | 1.18E-54 | 0.245898208 | 0.545 | 0.396 | 3.67E-50 | 1.278769399 |
| Tecr | 1.34E-54 | 0.231868712 | 0.329 | 0.198 | 4.17E-50 | 1.26095417 |
| Ndufa8 | 2.14E-54 | 0.242934697 | 0.407 | 0.267 | 6.65E-50 | 1.274985362 |
| Cd52 | 2.17E-54 | 0.187139883 | 0.974 | 0.94 | 6.72E-50 | 1.205795944 |
| Ubc | 3.65E-54 | 0.198055855 | 0.942 | 0.894 | 1.13E-49 | 1.21903048 |
| Basp1 | 4.47E-54 | -0.444789384 | 0.243 | 0.351 | 1.39E-49 | 0.640959258 |
| Timp2 | 7.56E-54 | -0.370984282 | 0.326 | 0.35 | 2.35E-49 | 0.690054788 |
| G0s2 | 8.02E-54 | -0.590066511 | 0.307 | 0.436 | 2.49E-49 | 0.554290417 |
| Rab5c | 1.46E-53 | 0.234944279 | 0.578 | 0.434 | 4.55E-49 | 1.264838289 |
| Cd300lf | 1.74E-53 | 0.236293385 | 0.755 | 0.631 | 5.40E-49 | 1.266545842 |
| Tspo | 2.28E-53 | 0.212741918 | 0.821 | 0.707 | 7.07E-49 | 1.237065345 |
| Tnfaip2 | 3.53E-53 | 0.256953515 | 0.844 | 0.746 | 1.10E-48 | 1.292985021 |
| Thbs1 | 5.36E-53 | -0.603100578 | 0.252 | 0.356 | 1.66E-48 | 0.547112638 |
| Prmt1 | 6.46E-53 | 0.182406752 | 0.175 | 0.078 | 2.01E-48 | 1.200102238 |
| Psmd8 | 7.03E-53 | 0.273390929 | 0.463 | 0.324 | 2.18E-48 | 1.314413987 |
| Arhgef1 | 7.45E-53 | 0.221344823 | 0.329 | 0.199 | 2.31E-48 | 1.24775361 |
| Egr1 | 9.27E-53 | -0.388603842 | 0.463 | 0.569 | 2.88E-48 | 0.678002813 |
| Rps2 | 1.66E-52 | 0.187418171 | 0.894 | 0.802 | 5.14E-48 | 1.206131549 |
| Hist2h2aa1 | 2.27E-52 | -0.257502307 | 0.079 | 0.165 | 7.05E-48 | 0.772979843 |
| Cxcr4 | 6.22E-52 | 0.258109104 | 0.621 | 0.479 | 1.93E-47 | 1.294480043 |
| Psme1 | 8.74E-52 | 0.256963192 | 0.587 | 0.448 | 2.72E-47 | 1.292997533 |
| Unc119 | 1.02E-51 | 0.2186406 | 0.32 | 0.194 | 3.17E-47 | 1.244383965 |
| Smim14 | 1.16E-51 | 0.228279758 | 0.445 | 0.304 | 3.61E-47 | 1.256436774 |
| Anxa5 | 1.43E-51 | -0.319059606 | 0.325 | 0.393 | 4.43E-47 | 0.726832224 |
| H2-Q4 | 2.31E-51 | 0.292911443 | 0.408 | 0.274 | 7.19E-47 | 1.34032409 |
| Ppib | 4.37E-51 | 0.219195013 | 0.62 | 0.479 | 1.36E-46 | 1.245074059 |
| Ptma | 8.42E-51 | 0.217086436 | 0.829 | 0.732 | 2.61E-46 | 1.24245149 |
| Midn | 9.01E-51 | 0.209660062 | 0.312 | 0.19 | 2.80E-46 | 1.233258757 |
| Sub1 | 9.91E-51 | 0.225300558 | 0.763 | 0.644 | 3.08E-46 | 1.252699168 |
| Ccl9 | 1.37E-50 | -0.370663971 | 0.129 | 0.231 | 4.24E-46 | 0.690275856 |
| Fam50a | 2.93E-50 | -0.260401369 | 0.191 | 0.265 | 9.09E-46 | 0.770742171 |
| S100a11 | 8.47E-50 | -0.297855126 | 0.908 | 0.909 | 2.63E-45 | 0.742408888 |
| Mar7 | 1.40E-49 | -0.227628508 | 0.527 | 0.563 | 4.36E-45 | 0.796420069 |
| Psma6 | 4.10E-49 | 0.216337104 | 0.441 | 0.304 | 1.27E-44 | 1.24152083 |
| Gm5150 | 5.18E-49 | -0.292300019 | 0.12 | 0.203 | 1.61E-44 | 0.746544525 |
| Lmna | 5.49E-49 | 0.366138108 | 0.439 | 0.307 | 1.70E-44 | 1.442154402 |
| AC163354.1 | 7.39E-49 | 0.197030243 | 0.102 | 0.032 | 2.30E-44 | 1.217780869 |
| Cd47 | 1.25E-48 | 0.218102642 | 0.784 | 0.671 | 3.88E-44 | 1.243714718 |
| Psma5 | 1.27E-48 | 0.220646954 | 0.407 | 0.274 | 3.96E-44 | 1.246883146 |
| Mdh1 | 1.41E-48 | 0.193388352 | 0.209 | 0.107 | 4.37E-44 | 1.21335391 |
| Tatdn2 | 1.47E-48 | 0.197294909 | 0.222 | 0.117 | 4.55E-44 | 1.218103217 |
| Rps21 | 2.53E-48 | -0.195703634 | 0.936 | 0.942 | 7.87E-44 | 0.822255887 |
| Ucp2 | 2.73E-48 | 0.213112596 | 0.665 | 0.53 | 8.47E-44 | 1.237523984 |
| Eif6 | 5.35E-48 | 0.20633334 | 0.387 | 0.257 | 1.66E-43 | 1.229162865 |
| Isg15 | 8.53E-48 | 0.324428991 | 0.293 | 0.175 | 2.65E-43 | 1.383240577 |
| Adssl1 | 9.49E-48 | 0.230463364 | 0.312 | 0.19 | 2.95E-43 | 1.259183335 |
| Gbp5 | 1.38E-47 | 0.283311602 | 0.215 | 0.112 | 4.27E-43 | 1.327518755 |
| Myd88 | 1.45E-47 | 0.196617287 | 0.354 | 0.229 | 4.51E-43 | 1.217278084 |
| St13 | 2.11E-47 | -0.252231694 | 0.335 | 0.391 | 6.55E-43 | 0.777064676 |
| Pitpna | 3.47E-47 | 0.219999749 | 0.644 | 0.514 | 1.08E-42 | 1.246076418 |
| Psmb9 | 5.29E-47 | 0.226618291 | 0.466 | 0.33 | 1.64E-42 | 1.25435098 |
| Fis1 | 5.72E-47 | 0.19698555 | 0.57 | 0.432 | 1.78E-42 | 1.217726444 |
| Kpna4 | 8.57E-47 | -0.237185961 | 0.314 | 0.367 | 2.66E-42 | 0.78884458 |
| Pcif1 | 2.11E-46 | 0.191325279 | 0.317 | 0.198 | 6.54E-42 | 1.210853254 |
| 2310001H17Rik | 2.15E-46 | -0.281650087 | 0.375 | 0.462 | 6.68E-42 | 0.754537661 |
| Itgb1 | 2.18E-46 | -0.246535873 | 0.309 | 0.363 | 6.77E-42 | 0.781503326 |
| Pnpla2 | 2.60E-46 | 0.197810706 | 0.379 | 0.253 | 8.08E-42 | 1.218731674 |
| Bcl2a1a | 2.73E-46 | -0.311652772 | 0.403 | 0.483 | 8.47E-42 | 0.732235737 |
| Rsad2 | 3.37E-46 | 0.354540436 | 0.235 | 0.13 | 1.05E-41 | 1.425525384 |
| Nt5c | 7.87E-46 | 0.197652094 | 0.236 | 0.131 | 2.44E-41 | 1.218538383 |
| Mitd1 | 1.03E-45 | 0.209109202 | 0.186 | 0.092 | 3.20E-41 | 1.232579591 |
| mt-Nd4l | 1.24E-45 | -0.252064061 | 0.249 | 0.303 | 3.85E-41 | 0.777194949 |
| Socs1 | 1.31E-45 | 0.256121335 | 0.219 | 0.117 | 4.07E-41 | 1.291909472 |
| Mrpl57 | 1.63E-45 | 0.205881948 | 0.385 | 0.258 | 5.05E-41 | 1.228608156 |
| Sec62 | 2.11E-45 | -0.22735262 | 0.453 | 0.509 | 6.54E-41 | 0.796639822 |
| Capzb | 2.94E-45 | 0.192993937 | 0.716 | 0.593 | 9.14E-41 | 1.21287544 |
| Mllt6 | 3.05E-45 | 0.237465269 | 0.23 | 0.126 | 9.46E-41 | 1.268030955 |
| Tmem219 | 2.21E-44 | 0.191196294 | 0.216 | 0.116 | 6.88E-40 | 1.210697081 |
| Lamtor4 | 2.56E-44 | 0.195244076 | 0.504 | 0.37 | 7.94E-40 | 1.215607651 |
| Odc1 | 3.17E-44 | -0.364956445 | 0.188 | 0.252 | 9.85E-40 | 0.694226888 |
| Mkrn1 | 5.07E-44 | 0.185739812 | 0.516 | 0.383 | 1.57E-39 | 1.204108925 |
| Lmnb1 | 5.31E-44 | -0.269251731 | 0.557 | 0.639 | 1.65E-39 | 0.763950922 |
| Rpl29 | 5.61E-44 | 0.194553252 | 0.824 | 0.73 | 1.74E-39 | 1.21476817 |
| Ccr1 | 5.84E-44 | -0.256675877 | 0.601 | 0.66 | 1.81E-39 | 0.77361892 |
| Gpx4 | 7.57E-44 | 0.21249977 | 0.783 | 0.681 | 2.35E-39 | 1.236765829 |
| Furin | 1.04E-43 | 0.225116982 | 0.399 | 0.273 | 3.23E-39 | 1.252469224 |
| Creg1 | 1.20E-43 | -0.319723463 | 0.497 | 0.526 | 3.72E-39 | 0.726349872 |
| Alcam | 1.27E-43 | -0.264403566 | 0.107 | 0.187 | 3.95E-39 | 0.767663674 |
| Litaf | 1.50E-43 | -0.196027577 | 0.716 | 0.741 | 4.65E-39 | 0.821989566 |
| Tgif1 | 3.37E-43 | 0.204857471 | 0.714 | 0.595 | 1.05E-38 | 1.227350119 |
| Slc31a1 | 3.41E-43 | 0.236664023 | 0.351 | 0.23 | 1.06E-38 | 1.267015358 |
| Osm | 8.39E-43 | -0.32983996 | 0.407 | 0.492 | 2.61E-38 | 0.719038799 |
| Icam1 | 1.71E-42 | 0.310957642 | 0.511 | 0.389 | 5.31E-38 | 1.364731412 |
| Hmgb2 | 1.88E-42 | -0.183616106 | 0.768 | 0.782 | 5.83E-38 | 0.83225524 |
| Slc43a2 | 4.26E-42 | 0.221304934 | 0.316 | 0.201 | 1.32E-37 | 1.247703839 |
| Ncor1 | 5.81E-42 | -0.209748779 | 0.403 | 0.448 | 1.80E-37 | 0.810787907 |
| Gm26532 | 6.09E-42 | 0.187192319 | 0.608 | 0.478 | 1.89E-37 | 1.205859172 |
| Supt4a | 6.99E-42 | -0.189423175 | 0.512 | 0.54 | 2.17E-37 | 0.827436282 |
| Tgoln1 | 7.56E-42 | -0.225720916 | 0.404 | 0.44 | 2.35E-37 | 0.797940763 |
| Rgcc | 1.77E-41 | 0.315306894 | 0.491 | 0.368 | 5.50E-37 | 1.370679899 |
| Gm19705 | 2.11E-41 | 0.184675747 | 0.125 | 0.051 | 6.56E-37 | 1.202828356 |
| mt-Nd1 | 3.12E-41 | 0.276695258 | 0.701 | 0.587 | 9.68E-37 | 1.318764427 |
| Prdx1 | 3.98E-41 | -0.271798945 | 0.601 | 0.604 | 1.24E-36 | 0.762007451 |
| Dmxl2 | 7.71E-41 | 0.231094171 | 0.501 | 0.374 | 2.39E-36 | 1.259977888 |
| Fosb | 7.77E-41 | -0.2833892 | 0.615 | 0.675 | 2.41E-36 | 0.753226575 |
| Ctnnbip1 | 8.36E-41 | 0.202474631 | 0.209 | 0.114 | 2.59E-36 | 1.224429023 |
| Mmp8 | 2.59E-40 | -0.378223488 | 0.247 | 0.348 | 8.03E-36 | 0.685077377 |
| Xpo1 | 3.84E-40 | -0.231104328 | 0.142 | 0.205 | 1.19E-35 | 0.793656661 |
| Lmo4 | 4.69E-40 | 0.230434785 | 0.482 | 0.356 | 1.46E-35 | 1.259147349 |
| Ankrd33b | 4.88E-40 | -0.295603962 | 0.251 | 0.339 | 1.51E-35 | 0.744082054 |
| Ptpn1 | 5.53E-40 | 0.220385869 | 0.589 | 0.464 | 1.72E-35 | 1.246557645 |
| Pilra | 6.84E-40 | -0.249323733 | 0.376 | 0.456 | 2.13E-35 | 0.779327638 |
| Lgals3bp | 7.58E-40 | 0.203340162 | 0.168 | 0.084 | 2.35E-35 | 1.225489262 |
| Itpr2 | 7.89E-40 | -0.624478489 | 0.24 | 0.281 | 2.45E-35 | 0.535540646 |
| Aoah | 8.62E-40 | 0.242063356 | 0.35 | 0.234 | 2.68E-35 | 1.273874898 |
| Ifrd1 | 1.00E-39 | -0.241245731 | 0.74 | 0.766 | 3.10E-35 | 0.785648544 |
| Psmb4 | 1.51E-39 | 0.190026886 | 0.531 | 0.405 | 4.70E-35 | 1.20928211 |
| Snap23 | 1.58E-39 | -0.201822041 | 0.482 | 0.52 | 4.89E-35 | 0.81724035 |
| Trem1 | 1.60E-39 | -0.256990406 | 0.555 | 0.612 | 4.96E-35 | 0.773375633 |
| Tuba4a | 2.57E-39 | 0.191065271 | 0.374 | 0.257 | 7.98E-35 | 1.210538463 |
| Kctd12 | 6.96E-39 | -0.286743093 | 0.348 | 0.401 | 2.16E-34 | 0.750704565 |
| Mef2d | 9.08E-39 | 0.188972068 | 0.367 | 0.252 | 2.82E-34 | 1.20800721 |
| Mat2a | 1.64E-38 | 0.232725651 | 0.441 | 0.321 | 5.09E-34 | 1.262035193 |
| H2-Q10 | 1.69E-38 | -0.319443515 | 0.07 | 0.138 | 5.24E-34 | 0.726553241 |
| Tnfaip3 | 1.86E-38 | 0.245041059 | 0.811 | 0.725 | 5.78E-34 | 1.277673772 |
| Tpm4 | 1.88E-38 | -0.258704833 | 0.314 | 0.385 | 5.82E-34 | 0.772050873 |
| Cd63 | 1.88E-38 | 0.20391532 | 0.746 | 0.637 | 5.83E-34 | 1.226194314 |
| Jaml | 2.01E-38 | -0.303721203 | 0.175 | 0.257 | 6.25E-34 | 0.738066609 |
| Trps1 | 2.06E-38 | -0.241367361 | 0.261 | 0.328 | 6.40E-34 | 0.785552992 |
| Batf | 2.45E-38 | 0.222656192 | 0.242 | 0.143 | 7.62E-34 | 1.24939095 |
| Dock10 | 2.85E-38 | 0.261366191 | 0.38 | 0.265 | 8.85E-34 | 1.298703152 |
| Pnp | 3.74E-38 | 0.2396963 | 0.505 | 0.384 | 1.16E-33 | 1.270863131 |
| Hsp90b1 | 4.92E-38 | -0.230434113 | 0.366 | 0.421 | 1.53E-33 | 0.79418876 |
| Tmem160 | 4.92E-38 | 0.199434934 | 0.24 | 0.143 | 1.53E-33 | 1.22071278 |
| B2m | 6.69E-38 | 0.207339999 | 0.983 | 0.982 | 2.08E-33 | 1.230400836 |
| Neurl3 | 1.26E-37 | -0.232548904 | 0.431 | 0.457 | 3.92E-33 | 0.792510991 |
| Rflnb | 2.02E-37 | 0.197394953 | 0.164 | 0.083 | 6.28E-33 | 1.218225088 |
| Actg1 | 1.82E-36 | -0.23158553 | 0.917 | 0.919 | 5.66E-32 | 0.793274843 |
| Ttc7 | 1.99E-36 | -0.221099664 | 0.148 | 0.21 | 6.19E-32 | 0.801636782 |
| Sys1 | 2.03E-36 | 0.197049074 | 0.421 | 0.304 | 6.30E-32 | 1.217803801 |
| Top1 | 2.26E-36 | -0.218631973 | 0.455 | 0.509 | 7.02E-32 | 0.803617417 |
| Tgfbi | 3.96E-36 | 0.234914167 | 0.674 | 0.562 | 1.23E-31 | 1.264800202 |
| Slc6a6 | 4.44E-36 | 0.186257468 | 0.503 | 0.383 | 1.38E-31 | 1.204732401 |
| Limd2 | 4.51E-36 | 0.209815079 | 0.358 | 0.247 | 1.40E-31 | 1.233449948 |
| Pycard | 5.67E-36 | 0.207271218 | 0.551 | 0.431 | 1.76E-31 | 1.23031621 |
| Dusp2 | 5.79E-36 | 0.285258139 | 0.401 | 0.29 | 1.80E-31 | 1.330105335 |
| Pcbd2 | 1.23E-35 | -0.213699454 | 0.133 | 0.199 | 3.82E-31 | 0.807591066 |
| Inhba | 1.26E-35 | -0.437840907 | 0.092 | 0.159 | 3.93E-31 | 0.645428458 |
| Nos2 | 1.68E-35 | 0.266125661 | 0.202 | 0.115 | 5.21E-31 | 1.304899023 |
| AC110211.1 | 1.84E-35 | -0.254027159 | 0.209 | 0.283 | 5.72E-31 | 0.775670735 |
| Ndufa11 | 1.85E-35 | 0.200828717 | 0.388 | 0.275 | 5.74E-31 | 1.222415375 |
| Fabp5 | 2.29E-35 | -0.23353917 | 0.401 | 0.43 | 7.12E-31 | 0.791726583 |
| St8sia4 | 4.62E-35 | -0.227278404 | 0.058 | 0.113 | 1.43E-30 | 0.796698947 |
| Ssh2 | 4.88E-35 | -0.237540584 | 0.342 | 0.405 | 1.52E-30 | 0.788564887 |
| Acta2 | 5.75E-35 | -0.207352205 | 0.053 | 0.106 | 1.79E-30 | 0.812733351 |
| Tbcb | 7.26E-35 | 0.182765819 | 0.349 | 0.24 | 2.25E-30 | 1.200533233 |
| Gpr84 | 1.96E-34 | 0.295450828 | 0.364 | 0.257 | 6.08E-30 | 1.343732014 |
| Psme2 | 2.16E-34 | 0.196509367 | 0.565 | 0.449 | 6.70E-30 | 1.217146722 |
| Parp14 | 1.39E-33 | 0.211894099 | 0.321 | 0.217 | 4.32E-29 | 1.236016983 |
| Aldh2 | 1.40E-33 | -0.253019062 | 0.231 | 0.268 | 4.36E-29 | 0.776453081 |
| Zfp36l2 | 2.47E-33 | 0.215288117 | 0.709 | 0.608 | 7.66E-29 | 1.240219173 |
| Syngr1 | 2.96E-33 | 0.21385896 | 0.157 | 0.082 | 9.19E-29 | 1.238447972 |
| Tsc22d3 | 2.98E-33 | 0.197339632 | 0.775 | 0.682 | 9.25E-29 | 1.218157696 |
| Steap4 | 3.60E-33 | -0.303293235 | 0.095 | 0.167 | 1.12E-28 | 0.738382545 |
| Ecm1 | 4.09E-33 | 0.189384803 | 0.18 | 0.1 | 1.27E-28 | 1.208505899 |
| Pgam1 | 4.16E-33 | 0.212658435 | 0.597 | 0.487 | 1.29E-28 | 1.236962076 |
| 4833407H14Rik | 4.61E-33 | -0.226714555 | 0.104 | 0.161 | 1.43E-28 | 0.797148292 |
| Cd80 | 5.88E-33 | -0.248812808 | 0.17 | 0.245 | 1.83E-28 | 0.779725919 |
| E030030I06Rik | 7.26E-33 | -0.196363944 | 0.164 | 0.188 | 2.25E-28 | 0.821713123 |
| Tmcc1 | 8.07E-33 | -0.243439743 | 0.293 | 0.349 | 2.51E-28 | 0.783926712 |
| Klf6 | 8.46E-33 | -0.220453169 | 0.626 | 0.652 | 2.63E-28 | 0.802155204 |
| Tgm2 | 1.00E-32 | -0.232243892 | 0.336 | 0.38 | 3.11E-28 | 0.792752753 |
| Rilpl2 | 1.58E-32 | 0.226995334 | 0.409 | 0.301 | 4.91E-28 | 1.254824013 |
| Samsn1 | 3.70E-32 | -0.188068722 | 0.706 | 0.757 | 1.15E-27 | 0.828557765 |
| Lsp1 | 4.48E-32 | -0.195577623 | 0.607 | 0.595 | 1.39E-27 | 0.822359507 |
| Snrpf | 1.33E-31 | 0.212663754 | 0.41 | 0.303 | 4.14E-27 | 1.236968655 |
| Gcnt2 | 2.65E-31 | -0.263444502 | 0.265 | 0.343 | 8.22E-27 | 0.768400266 |
| Srgn | 4.54E-31 | -0.186340766 | 0.932 | 0.95 | 1.41E-26 | 0.829990714 |
| Dusp1 | 8.81E-31 | -0.214054517 | 0.957 | 0.963 | 2.73E-26 | 0.807304372 |
| Slpi | 2.91E-30 | -0.227911375 | 0.758 | 0.781 | 9.05E-26 | 0.79619482 |
| Gadd45a | 3.75E-30 | -0.270977486 | 0.397 | 0.474 | 1.17E-25 | 0.762633666 |
| Chd7 | 6.98E-30 | -0.199242047 | 0.407 | 0.429 | 2.17E-25 | 0.819351548 |
| Hist1h4h | 2.75E-29 | -0.280224424 | 0.084 | 0.138 | 8.53E-25 | 0.755614145 |
| Rnf144a | 1.27E-28 | -0.197634436 | 0.151 | 0.201 | 3.95E-24 | 0.820669806 |
| Hdc | 1.46E-28 | -0.269973511 | 0.512 | 0.592 | 4.53E-24 | 0.763399716 |
| Cd48 | 1.82E-28 | 0.189541563 | 0.202 | 0.123 | 5.65E-24 | 1.20869536 |
| Psmb6 | 2.98E-28 | 0.182663506 | 0.471 | 0.366 | 9.25E-24 | 1.200410409 |
| Nabp1 | 3.54E-28 | -0.215969126 | 0.223 | 0.268 | 1.10E-23 | 0.805760179 |
| Cd300ld | 7.06E-28 | -0.25818867 | 0.203 | 0.274 | 2.19E-23 | 0.77244948 |
| 1600014C10Rik | 1.11E-27 | 0.183478482 | 0.293 | 0.201 | 3.43E-23 | 1.201389114 |
| Ptgs2os2 | 1.31E-27 | -0.224652933 | 0.107 | 0.166 | 4.07E-23 | 0.798793405 |
| Ddt | 1.48E-27 | -0.223755497 | 0.167 | 0.18 | 4.59E-23 | 0.799510593 |
| Cybb | 3.23E-27 | -0.245652224 | 0.44 | 0.468 | 1.00E-22 | 0.782194206 |
| Ifit1 | 1.93E-26 | 0.211108957 | 0.109 | 0.053 | 6.00E-22 | 1.235046914 |
| Eea1 | 4.22E-26 | -0.211116863 | 0.176 | 0.213 | 1.31E-21 | 0.80967944 |
| Mcfd2 | 8.74E-26 | -0.193054052 | 0.145 | 0.162 | 2.71E-21 | 0.82443741 |
| Rgs10 | 2.99E-25 | 0.189530888 | 0.279 | 0.194 | 9.30E-21 | 1.208682457 |
| Rnd1 | 3.31E-25 | 0.208372374 | 0.215 | 0.138 | 1.03E-20 | 1.231671727 |
| Stx11 | 3.75E-25 | -0.207627126 | 0.277 | 0.328 | 1.16E-20 | 0.812509944 |
| Mt1 | 7.76E-25 | 0.359643634 | 0.417 | 0.327 | 2.41E-20 | 1.432818716 |
| Mdm2 | 1.50E-24 | -0.209677619 | 0.43 | 0.415 | 4.67E-20 | 0.810845605 |
| Nampt | 1.77E-24 | 0.192622431 | 0.399 | 0.305 | 5.51E-20 | 1.212424933 |
| Fam107b | 2.73E-24 | -0.188199752 | 0.279 | 0.319 | 8.48E-20 | 0.828449206 |
| Pi16 | 6.02E-24 | -0.206315867 | 0.136 | 0.184 | 1.87E-19 | 0.813576054 |
| Plbd1 | 1.48E-23 | -0.192731761 | 0.367 | 0.4 | 4.60E-19 | 0.824703162 |
| Diaph2 | 1.93E-23 | -0.185855997 | 0.172 | 0.219 | 5.98E-19 | 0.830393165 |
| Gm17056 | 5.08E-23 | -0.204828996 | 0.108 | 0.159 | 1.58E-18 | 0.814786636 |
| Rdh12 | 1.24E-22 | -0.200642471 | 0.222 | 0.305 | 3.87E-18 | 0.818204912 |
| Ifi27l2a | 1.28E-22 | -0.304999753 | 0.105 | 0.167 | 3.99E-18 | 0.737123557 |
| Trim30b | 1.96E-22 | -0.203406157 | 0.219 | 0.257 | 6.10E-18 | 0.815946772 |
| Dck | 3.86E-22 | -0.187839585 | 0.074 | 0.121 | 1.20E-17 | 0.82874764 |
| Ddx3y | 3.90E-22 | -0.197812088 | 0.361 | 0.403 | 1.21E-17 | 0.820524025 |
| Ccl3 | 9.36E-22 | -0.302235359 | 0.675 | 0.721 | 2.91E-17 | 0.739164075 |
| Ccrl2 | 1.50E-21 | 0.207968385 | 0.905 | 0.865 | 4.66E-17 | 1.231174246 |
| Cwc25 | 2.30E-21 | -0.250302758 | 0.253 | 0.289 | 7.15E-17 | 0.778565031 |
| Mmp9 | 4.09E-21 | -0.242834657 | 0.372 | 0.443 | 1.27E-16 | 0.784401198 |
| Zfhx3 | 9.19E-21 | -0.190312769 | 0.182 | 0.234 | 2.85E-16 | 0.826700527 |
| Fgl2 | 1.44E-20 | -0.184901138 | 0.306 | 0.32 | 4.47E-16 | 0.831186453 |
| Ninj1 | 1.49E-20 | 0.192908437 | 0.658 | 0.576 | 4.63E-16 | 1.212771744 |
| Cxcl10 | 1.61E-20 | 0.34563932 | 0.235 | 0.163 | 5.01E-16 | 1.412892922 |
| Clec4n | 2.75E-20 | -0.201567015 | 0.443 | 0.472 | 8.53E-16 | 0.817448795 |
| Cstb | 1.29E-19 | -0.260778313 | 0.824 | 0.808 | 4.00E-15 | 0.7704517 |
| Adam8 | 3.95E-19 | -0.198593954 | 0.282 | 0.346 | 1.23E-14 | 0.819882736 |
| Ccl4 | 5.45E-19 | -0.375880111 | 0.406 | 0.455 | 1.69E-14 | 0.686684654 |
| Fos | 3.14E-18 | -0.182986017 | 0.926 | 0.925 | 9.74E-14 | 0.8327798 |
| Cxcl3 | 7.89E-18 | -0.387102057 | 0.13 | 0.168 | 2.45E-13 | 0.679021792 |
| Cytip | 2.28E-17 | -0.182647587 | 0.404 | 0.449 | 7.09E-13 | 0.833061686 |
| Eprs | 4.16E-16 | -0.199218214 | 0.156 | 0.181 | 1.29E-11 | 0.819371075 |
| Ifitm6 | 4.55E-16 | 0.36908664 | 0.196 | 0.155 | 1.41E-11 | 1.446412915 |
| Gm5416 | 7.57E-16 | -0.267383161 | 0.069 | 0.106 | 2.35E-11 | 0.765379752 |
| Stfa2l1 | 1.49E-15 | 0.37184311 | 0.418 | 0.371 | 4.63E-11 | 1.45040541 |
| Il1rn | 2.55E-15 | -0.19195607 | 0.637 | 0.677 | 7.92E-11 | 0.825343125 |
| Plac8 | 1.29E-13 | 0.21431308 | 0.365 | 0.297 | 4.01E-09 | 1.239010503 |
| Gstm1 | 4.53E-13 | -0.216164573 | 0.189 | 0.219 | 1.41E-08 | 0.805602711 |
| Ccl2 | 1.17E-10 | -0.251753957 | 0.236 | 0.25 | 3.62E-06 | 0.777435997 |
| Il1a | 3.00E-05 | 0.23371319 | 0.428 | 0.406 | 0.93 | 1.263282119 |
